# Live Cell Imaging Reveals pH Oscillations in *Saccharomyces cerevisiae* During Metabolic Transitions

**DOI:** 10.1038/s41598-017-14382-0

**Published:** 2017-10-24

**Authors:** Benjamin J. T. Dodd, Joel M. Kralj

**Affiliations:** 1 BioFrontiers Institute, University of Colorado, Boulder, 80303 USA; 2 Molecular Cellular and Developmental Biology Department, University of Colorado, Boulder, 80303 USA

## Abstract

Addition of glucose to starved *Saccharomyces cerevisiae* initiates collective NADH dynamics termed glycolytic oscillations. Numerous questions remain about the extent to which single cells can oscillate, if oscillations occur in natural conditions, and potential physiological consequences of oscillations. In this paper, we report sustained glycolytic oscillations in single cells without the need for cyanide. Glucose addition to immobilized cells induced pH oscillations that could be imaged with fluorescent sensors. A population of cells had oscillations that were heterogeneous in frequency, start time, stop time, duration and amplitude. These changes in cytoplasmic pH were necessary and sufficient to drive changes in NADH. Oscillators had lower mitochondrial membrane potentials and budded more slowly than non-oscillators. We also uncovered a new type of oscillation during recovery from H_2_O_2_ challenge. Our data show that pH in *S. cerevisiae* changes over several time scales, and that imaging pH offers a new way to measure glycolytic oscillations on individual cells.

## Introduction

Single celled organisms experience a variety external conditions and must adapt their physiology to optimally perform in the new environment. Changing conditions often result in changes to cytoplasmic pH^1–3^ which can have significant effects on cellular processes including enzymatic activity, structural components, and proliferation^4^. Recently, several papers demonstrate that *Saccharomyces cerevisiae* leverage cytoplasmic pH as a regulator for gene transcription^5^, GPCR activation^6^, chromatin structure^7^, and PKA activity^8^. Given the fundamental role of pH in controlling many aspects of physiology, it is critical to understand the conditions in which single cells display dynamic changes in cytoplasmic pH.

One well documented cellular response in *S. cerevisiae* is a population level oscillation of NADH concentration in response to glucose and cyanide termed glycolytic oscillations^9,10^. Though the phenomenon has been experimentally and theoretically explored a the population level^11^, many important questions remain including (i) if single cells can undergo sustained oscillations, (ii) do oscillations occur in natural conditions, and (iii) what are the downstream consequences of oscillations^12^, if any^13^? Glycolytic oscillations are modeled as dynamic flux through glycolysis via ATP dependent inhibition of Phosphofructokinase (PFK)^14^, though it has been suggested that there are additional critical components^13,15^. How these other components are regulated remains unknown. New fluorescent biosensors with high sensitivity would enable studying glycolytic oscillations at the single cell level, and could reveal new biological functions of the dynamic response. Furthermore, a better understanding of glycolytic oscillations in yeast could help elucidate the function and regulation of similar oscillations in beta cells^16^ and muscle cells^17^, and how glycolytic dysregulation can lead to disease^18^.

In this paper, we report that *S. cerevisiae* undergo sustained single cell glycolytic oscillations without the need for cyanide. The pH changes during these oscillations can be imaged with genetically encoded sensors and correspond with oscillations of cytoplasmic NADH and mitochondrial membrane potential. The observed amplitude of pH oscillations is within the range reported to have biologically relevant impacts on numerous cellular processes. We found changes in cytoplasmic pH were necessary and sufficient to drive corresponding changes in cytoplasmic NADH concentration. The duration of an oscillation is correlated with increased time to bud emergence in single cells. Finally, long term observations of cytoplasmic pH led to the discovery of a new class of oscillation during challenge with H_2_O_2_ occurring on a timescale 10× longer than typical glycolytic oscillations. Overall, our experimental results answer some long standing questions regarding glycolytic oscillations, as well as provide strategies to study metabolic oscillations in yeast across various contexts.

## Results

**Figure 1:**
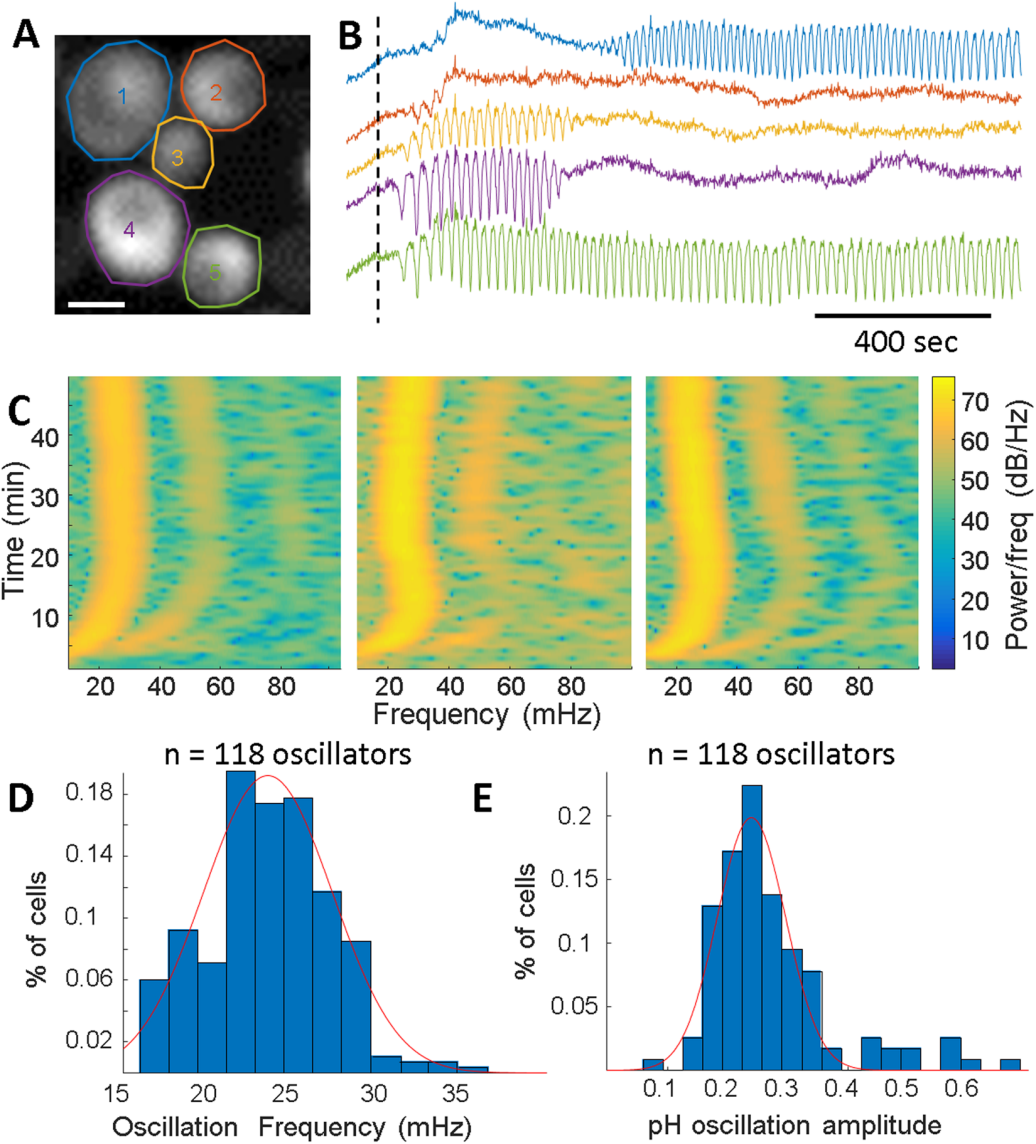
Individual *S. cerevisiae* showed pH oscillations upon glucose addition. (**A**) Image of cells expressing SEP with user selected ROIs. Scale bar is 2 μm. (**B**) SEP fluorescence time traces from each ROI in (**A**). Glucose addition (5 mM) is indicated by the dashed line. (**C**) Spectrograms for oscillations arising from individual cells. The change in frequency is seen by the changing dominant peak. (**D**) Frequency distribution from a population of cells in the same field of view. The distribution is fit to a Gaussian centered at 0.022 Hz (45 second period). (**E**) pH oscillation amplitude distribution from a population of cells in the same field of view. The distribution is fit to a Gaussian centered at 0.28 pH units.

To observe changes in cytoplasmic pH, *S. cerevisiae* were transformed with a plasmid expressing a pH sensitive GFP derivative, super ecliptic pHluorin^19^ (SEP, Fig. 1A). Cells were grown to diauxic shift (OD ~2.8) in synthetic dropout medium, immobilized to a glass coverslip using a 1% agarose pad. Upon glucose addition, cytoplasmic pH oscillations were observed (Fig. 1B). pH oscillations were unsynchronized between adjacent cells, and synchronization was not observed within 1 hour of oscillations initiation (Fig. S1). Within single cells, the oscillating pH showed no spatial heterogeneity when sampled at 10 Hz (Fig. S2), consistent with rapid diffusion of protons in the cytoplasm^20^. The oscillation initiation time, stop time, and duration was heterogeneous throughout the population (Fig. S3). Cells showed heterogeneity in initial SEP intensity, but these values were uncorrelated with oscillations and are likely due to stochastic variations in sensor expression (Fig. S3). We observed oscillation termination and re-initiation in cells within a single experiment (Fig. S4). Both budded and non-budded cells displayed oscillations, with the daughter bud oscillations in phase with the mother cell (Fig. S5). Glucose addition to cells grown to late log phase (OD ~1.0) or to cells that had already terminated an oscillation phase did not induce oscillations (Fig. S6). The percentage of oscillating cells and median oscillation frequency increased as a function of increasing external glucose concentration moving from 0.014 Hz to 0.021 Hz with a 50 fold increase external glucose (Fig. S6). The density of cells under the agarose pad did not affect cells ability to oscillate as both isolated and 2D packed cells were able to undergo oscillations (Fig. S7). pH oscillations were measured using other genetically encoded fluorescent pH sensors (ratiometric pHluorin^21^ and pHuji^22^), as well as a pH sensitive organic dye (SNARF-5F) (Fig. S8), to confirm the observed dynamics were cytoplasmic pH oscillations.

To measure the frequency of pH oscillations throughout an experiment, spectrograms were created for oscillating *S. cerevisiae* by subtracting the mean fluorescence intensity, then taking sliding window Fourier transform across time (Fig. 1C). Higher order harmonics were observed in many cells and demonstrated fluorescent oscillations are not purely sinusoidal, though this could be due to cellular responses or a non-linear response of the sensor. The spectrograms showed that the fundamental frequency of a given cell could change with time, moving faster or slower. The mean cellular fundamental frequency within a population was distributed around a mean of 22 mHz (45 second period) with a fitted full width half max of 5 mHz (Fig. 1D). The mean population frequency varied from day-to-day resulting in mean population level frequencies ranging from 0.2 to 0.35 Hz from currently unknown variables.

Glycolytic oscillations have canonically been induced through the sequential addition of glucose and cyanide to starved cells, resulting in the induction of synchronized oscillations^23^. We tested addition of 5 mM KCN to a population of pre-induced oscillating, unsynchronized cells on an agarose pad (Fig. S9). Upon addition of cyanide, all cells terminated oscillations and increased pH upon exposure to the cyanide solution. A smaller fraction of cells began oscillations ~12 minutes post-cyanide exposure. Cells oscillating both before and after cyanide treatment had a lower frequency and smaller pH amplitude relative to pre-treatment. We observed no induction of synchronization between neighboring cells or within the entire population.

To record absolute cytoplasmic pH in intact *S. cerevisiae*, we a engineered a fusion protein between SEP and mRuby2^24^ expressed in the cytoplasm (Fig. S10). mRuby2 has a reported pKa of 5.3, and thus acts as an expression control for these experiments. Absolute values were calculated using the ratio of SEP to mRuby emission excited with 488 nm and 561 nm light, respectively, and fit to a calibration curve (Fig. S9). The calibration parameters were created by fitting individual cell’s response to the specified pH, followed by fitting the distribution of the population (Fig. S10). The best fit parameters were then applied to individual cells at a specific pH (pH 7) to generate confidence bounds of single cell absolute pH within +/− 0.18 units. Despite the uncertainty in absolute pH, the sensor can track dynamics within a single cell with high accuracy. The SEP-mRuby2 biosensor reported cytoplasmic pHs from a range of 6.6 to 8.1. The engineered biosensor was critical to measure sustained oscillations as UV and blue light depressed the pH oscillations (Fig. S11), preventing use of ratiometric pHluorins which require 395 nm excitation^25^. Light in this range disrupts the electron transport chain and the enzymatic reactivity of NADH^26–28^. Quantitative pH measurements showed oscillation amplitudes peaked at 0.28 pH units with a full width half maximum of 0.17 units (Fig. 1E). This amplitude of single cell pH oscillations was large enough to influence several known pH signaling pathways in *S. cerevisiae* ^5–7^.

**Figure 2:**
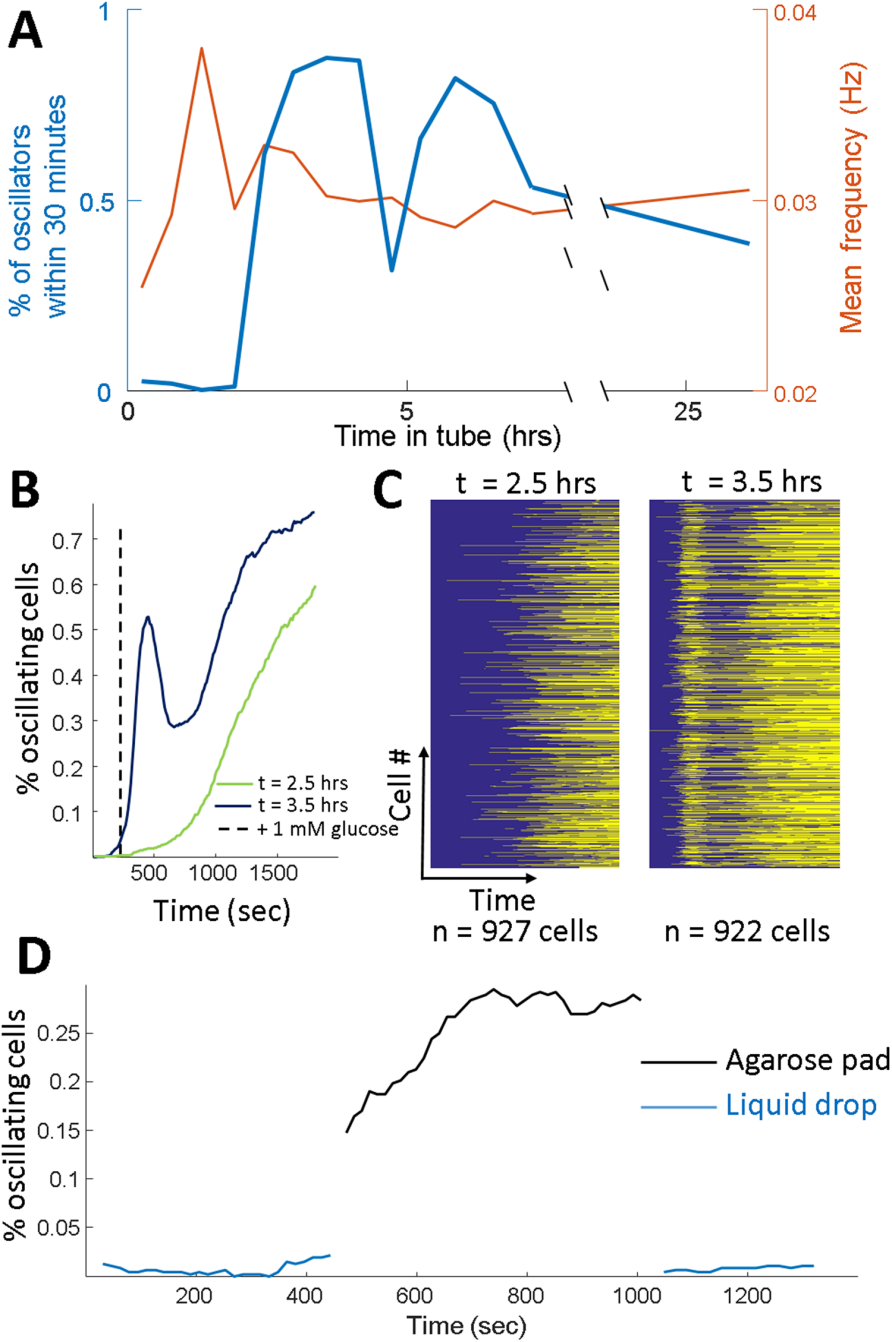
Conditions to generate pH oscillations. (**A**) Time course measuring the percentage of oscillating cells (blue) and the frequency of oscillating cells (red) as a function of the time in a closed tube. (**B**) Within a single 30 minute movie, the fraction of oscillating cells as a function of time after 2.5 hours (green) and 3.5 hours (purple) in a closed tube. (**C**) Individual cells either oscillating (yellow) or not oscillating (blue) as a function of time after glucose addition. Time points correspond to the lines in (**B**). (**D**) The same batch of cells were placed in a liquid drop or agarose pad and glucose was added simultaneously. Cells in the liquid drop had low numbers of oscillators, while the agarose pad induced a large fraction of oscillators up to 35%.

Sustained glycolytic oscillations were not present in cells collected in log or stationary phase from liquid cultures, and appeared only in cells collected in a narrow growth range making it challenging to replicate from day to day. Therefore, we sought to determine conditions to reliably generate populations containing large fractions of cells that could be induced into a sustained oscillatory state. We identified parameters that allowed for robust induction of sustained pH oscillations independent of cyanide. Cells were grown to an OD600 of ~1.0 and placed into a closed microcentrifuge tube. Cells sedimented to the bottom of the tube within 45 minutes, at which time measurements were initiated every 30 minutes. The fraction of oscillating cells, mean frequency, and start times were measured as a function of time post-sample collection. Within the first hour, few cells oscillated during a 30 minute movie. The percent of oscillators increased to a maximum of 91% within 3 hours after settling in the tube. After 25 hours in the closed tube, the fraction of oscillating cells within 30 minutes of glucose addition reduced to 35% off cells (Fig. 2A). Increasing time in the closed tube also resulted in increased steady state pH changes from baseline (Fig. S12), indicating increased cytoplasmic acidification due to starvation^1^. Unexpectedly, the glucose concentration in the supernatant was not depleted during the induction of oscillators, reducing from 110 to 50 mM, though local concentration near the pellet remains unknown. Oscillation initiation changed throughout the time course with the first measured oscillation starting earlier after the addition of glucose as the time course progressed (Fig. 2B,C). The mean frequency was stable with a small decrease from 32 to 29 mHz. Cells taken from log phase and spun down in the centrifuge showed a similar time course (Fig. S13). Additionally, yeast colonies grown on agar plates could be induced to oscillate upon glucose addition, though never with as high a fraction of oscillators (Fig. S14). The low numbers of oscillators in the colony could be due to a sub-population of differentiated cells^29^.

The experimental conditions we employed were distinct from traditional bulk glycolytic oscillation measurements. We used an agarose pad to immobilize the cells and sustained oscillations required solely glucose addition. Based on previous work demonstrating that *S. cerevisiae* immobilization led to enhanced glycolysis and slowed biomass accumulation^30^ and observations of glycolytic oscillations of hydrogel encased yeast^31^, we tested if hydrogel immobilization was essential for the observed oscillations. Cells were grown to log phase, pelleted, and left in a closed tube for 6 hours to ensure oscillating cells. Cells taken from the same batch in a liquid drop with no hydrogel did not show any oscillations upon glucose addition, while those under a hydrogel did oscillate as expected (Fig. 2C). Pads made from 3% agarose and 10% gelatin also induced oscillations and confirmed it was a mechanical and not chemical response (Fig. S15). This data suggested that the local mechanical environment is critical to initiation of oscillations, and may be related to the altered metabolic properties of immobilized *S. cerevisiae*.

**Figure 3:**
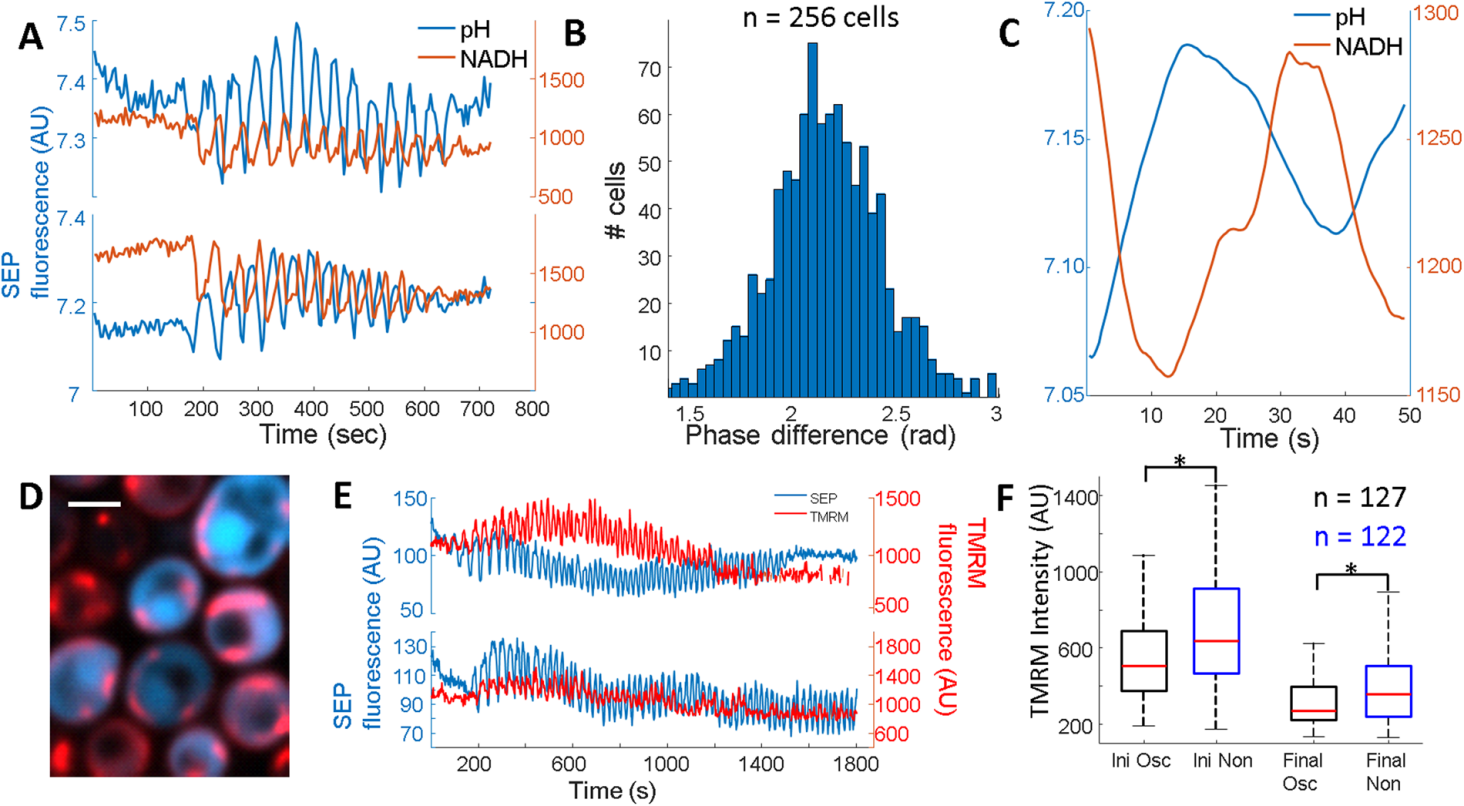
NADH and mitochondrial potential oscillate during cytoplasmic pH oscillations. (**A**) Time traces from 2 individual cells imaged with SEP-mRuby (pH) and NADH autofluorescence. (**B**) Phase difference between the pH oscillations and NADH oscillations. Each member of the histogram is the average phase difference from a single cell. (**C**) Average of many oscillations from a single cell in both pH (blue) and NADH (red) showing non-sinusoidal nature of the NADH autofluorescence. (**D**) Merged image showing cytoplasmic SEP (blue) and TMRM (red). Scale bar is 2 μm. (**E**) Time traces from 2 individual cells showing oscillations in pH (blue) and mitochondrial membrane potential (red). (**F**) Box plot of the TMRM intensity in mitochondria from oscillating (black boxes) and non-oscillating (blue boxes) cells from the same experiment before and 30 minutes after the addition of glucose. Red line indicates median, box indicates 75/25%, and black dashed lines indicate 90/10% of the maxima. Outliers were analyzed but not shown. *Represents a p value < 0.01 measured by an unpaired student t-test with unequal variance.

The observed pH oscillations possessed many characteristics of glycolytic oscillations typically induced in batch cultures. To confirm the observed pH oscillations were indeed glycolytic oscillations, cells expressing SEP-mRuby were immobilized with agarose and excited with 488 nm (SEP), 561 nm (mRuby) and 375 nm (NADH autofluorescence) light (Fig. 3A). NADH measurements were performed with the minimum possible light exposure given the UV suppression of oscillations in both WT and SEP expressing cells (Fig. S11). Extracted fluorescence traces show pH and NADH both oscillate in single cells, and they have an average phase difference of 2.1 radians (Fig. 3B). No cells were observed in which either signal oscillated independently. The phase difference of the pH and NADH signal was uncorrelated with oscillation frequency (PCC = 0.15, Fig. S16). No correlation was measured between the absolute pH oscillation amplitude and the NADH oscillation amplitude (PCC = 0.014, Fig. S16). NADH autofluorescence had a small decrease coincident with the start of the pH decrease (Fig. 3C) and was consistent across many cells.

The coupling between glycolysis and mitochondrial potential has been studied in populations of *S. cerevisiae* ^32,33^, so we sought to image single cell pH oscillations while simultaneously recording mitochondrial membrane potential. Cells were pre-incubated with tetramethylrhodamine methyl ester (TMRM), a cell permeant dye sequestered by polarized mitochondria. Simultaneous imaging of SEP and TMRM confirmed incorporation and spectral separation (Fig. S17). Glucose addition triggered cytoplasmic pH oscillations with corresponding mitochondrial TMRM signal oscillations in phase with the pH signal (Fig. 3D,E). Other groups have seen similar mitochondrial oscillations^34^. Every cell with an oscillating cytoplasmic pH showed oscillating TMRM. Targeting SEP-mRuby to the mitochondrial matrix showed oscillations of matrix pH, consistent with the changes in membrane potential (Fig. S17). Upon addition of glucose, both non-oscillators and oscillators showed decreased mitochondrial membrane potential consistent with glucose induced respiration repression. However, oscillators had lower TMRM staining compared to non-oscillators both before glucose, and afterwards (Fig. 3F). Oscillating cells therefore had lower mitochondrial membrane potential.

**Figure 4:**
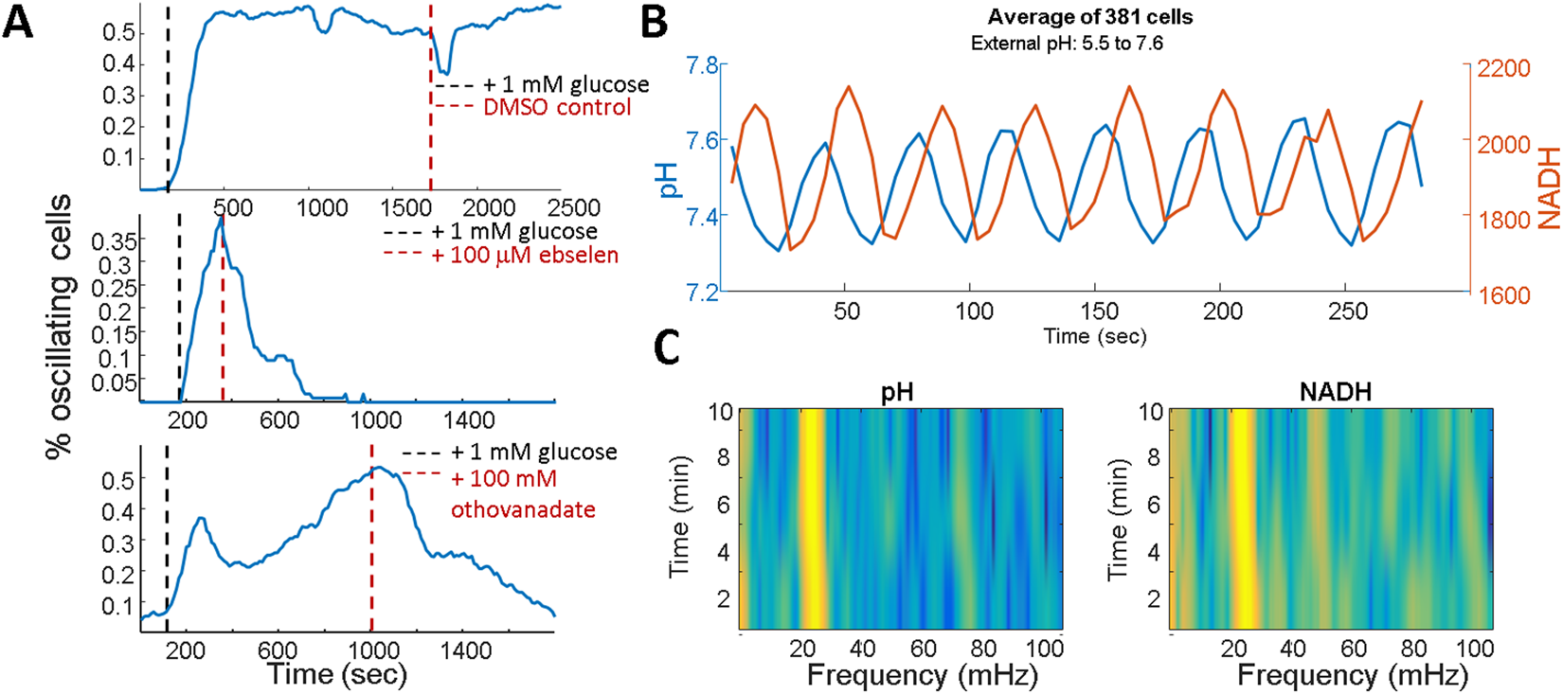
pH oscillations are necessary and sufficient to drive NADH oscillations. (**A**) Fraction of oscillating cells after the addition of glucose (black dashed line) a DMSO control (top), ebselen (middle) or sodium orthovandate (bottom) shown with red dashed lines. (**B**) Average time trace from a population of cells while recording pH and NADH measured on glass adhered cells with glucose while alternating external pH between 5.5 and 7.6 every 20 seconds. (**C**) Spectrograms of pH and NADH from the same experiment.

Processing glucose through the glycolytic pathway acidifies the cytoplasmic environment, but several possibilities exist for proton extrusion from the cytoplasm. Potential proton sinks include organelles, such as the vacuole, or the extracellular medium through the plasma membrane ATPase. Vma2 is a subunit of the vacuolar ATPase responsible for acidifying the vacuole which would lead to a corresponding basification of the cytoplasm. A Vma2 knockout strain still demonstrated oscillations (Fig. S18) suggesting the vacuole does not act as a proton sink during pH oscillations. Pma1, the plasma membrane ATPase, is an essential gene considered to be a major regulator of cytoplasmic pH by extruding protons from the cytoplasm through the plasma membrane to the exterior of the cell. We tested the potential role PMA1p might play in oscillations by the addition of small molecule inhibitors ebselen (100 μM) and sodium orthovanadate (100 mM), both of which stopped pH oscillations (Fig. 4A,B). Addition of 10 μM ebselen increased both the frequency and number of oscillating cells (Fig. S19). Based on this data, we hypothesized PMA plays a critical role in generating glycolytic oscillations by removing protons from the cytoplasm and increasing the efficiency of the glycolytic pathway via pH regulation^35^.

In light of the cooresponding changes in cytoplasmic pH, mitochondrial matrix pH, mitochondrial potential, and NADH, we sought to determine if changes in cytoplasmic pH could act as a master regulator of NADH. To test this hypothesis, we created a flow cell to change the external pH every 20 seconds from 5.2 to 7.6 in the presence of external glucose. During the flow, we monitored absolute cytoplasmic pH through SEP-mRuby fluorescence as well as NADH autofluorescence. Upon initiation of the external pH changes, we saw corresponding changes in cytoplasmic pH (Fig. 4C,D) with the population mean pH amplitude of 0.29 units, recapitulating the pH amplitudes we observed in glucose induced oscillations. The synthetically imposed pH oscillations led to corresponding NADH oscillations as observed in glucose induction (Fig. 4C,D). Combined with the PMA inhibitors, our data show that oscillating cytoplasmic pH is both necessary and sufficient to generate glycolytic oscillations.

**Figure 5:**
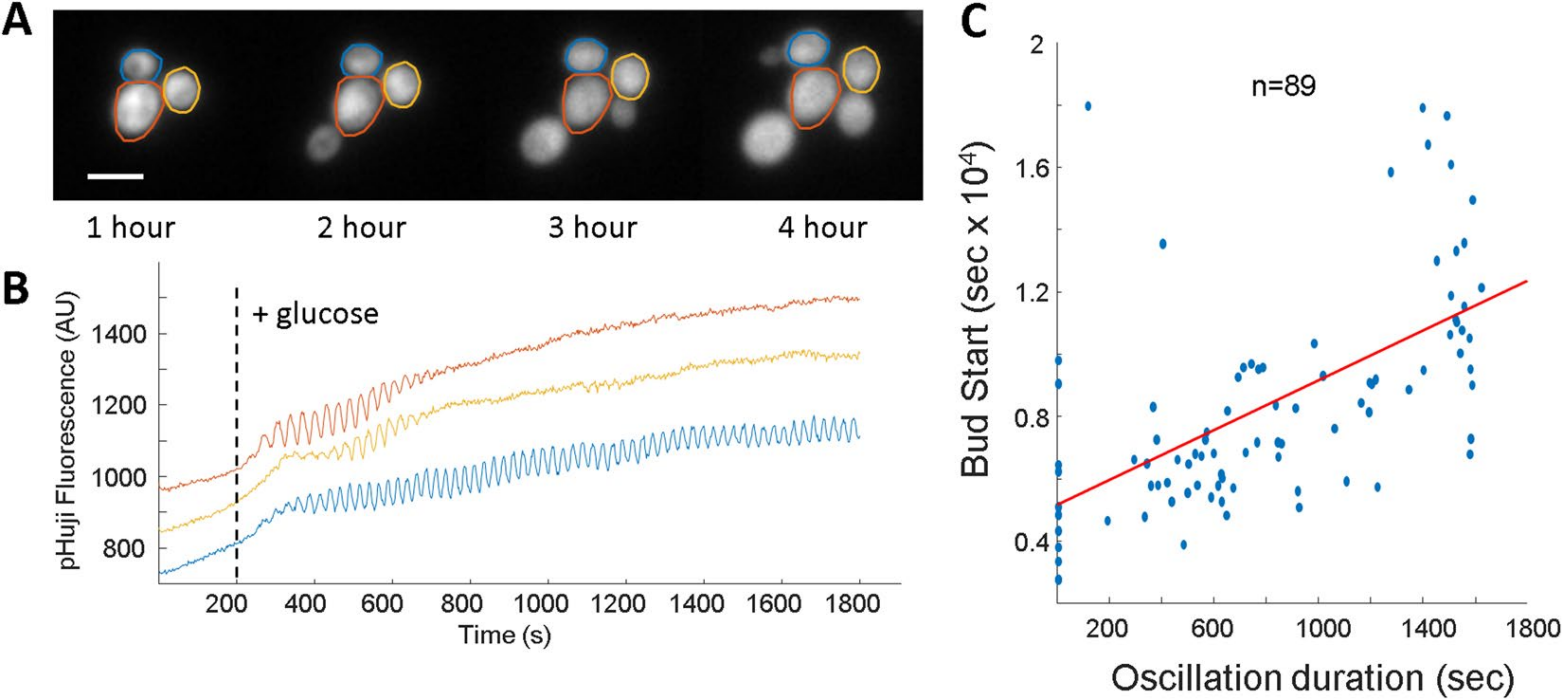
Oscillation duration is correlated with increased bud time. (**A**) Strip chart of cells expressing pHuji after exposure to glucose at time t = 0. Each cell buds, though the buds appear at different times. Scale bar is 3 μm. (**B**) Oscillations measured by pHuji fluorescence of the same cells in (**A**) after the addition of glucose. (**C**) Scatter plot showing the oscillation duration and emergence of the first bud. Oscillation duration is capped at 30 minutes as the imaging was slowed to once every 30 seconds to capture budding events. The red line indicates the best fit straight line to the data.

The observed glycolytic oscillations were not induced with cyanide and single cells within a population showed substantial heterogeneity in oscillation duration and frequency. We sought to leverage this heterogeneity to ask questions about the role of glycolytic oscillations affecting downstream cellular physiology by comparing oscillators and non-oscillators within a single experiment. To measure long term pH dynamics, pHuji was used to minimize phototoxicity as 561 nm light has much lower photoxicity than 488 nm^36^. pHuji expressing cells were placed under an agarose pad, and fed glucose followed by a 30 minute movie to identify oscillators. A longer, lower sampling rate movie was taken of the same cells over an additional 6 hours to look for budding events (Fig. 5A,B). Cell oscillation duration was compared to the time of the first budding event after glucose addition. We identified a strong correlation (PCC = 0.61) between the duration of oscillation and the time to first bud (Fig. 5C). Bud time was not correlated with initial pHuji intensity (Fig. S20).

**Figure 6:**
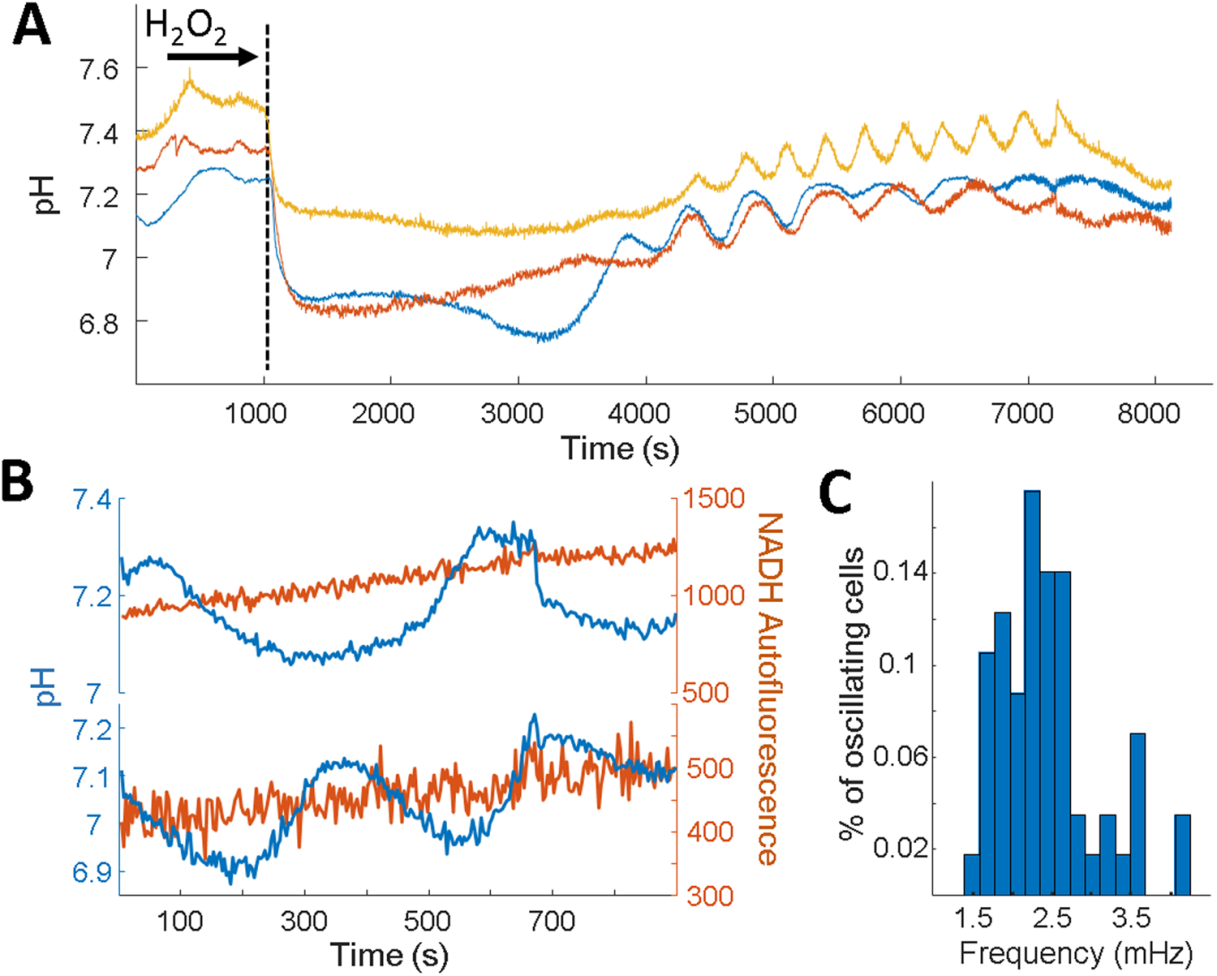
H_2_O_2_ induces slow pH oscillations. (**A**) Time traces of 3 individual cells expressing SEP-mRuby after the addition of glucose and hydrogen peroxide. (**B**) Time traces of 2 individual cells during the slow oscillations while imaging SEP-mRuby and NADH autofluorescence simultaneously. (**C**) Distribution of frequencies after H_2_O_2_ addition. The distribution is peaked at 2.4 mHz (416 second period).

The pH oscillations observed were associated with the transition from the metabolic states of respiration (low glucose) to glycolysis (high glucose). We hypothesized oscillations could be more a more general cellular mechanism during changes in metabolism. H_2_O_2_ exposure inhibits GAPDH activity^37^ and triggers *S. cerevisiae* to transition carbohydrate flux from glycolysis to regeneration of NADPH^38^. Cells expressing SEP-mRuby were grown to late-log and imaged under an agarose pad. Glucose addition did not induce glycolytic oscillations as expected from the time course data (Fig. 6A). After 15 minutes, H_2_O_2_ was added on top of the pad. Cells responded within 2 minutes by a rapid acidification of the cytoplasm consistent with previous results^39^. Cells remained acidic for ~30 minutes, after which cytoplasmic pH gradually increased. During this recovery, some cells showed clear oscillations (Fig. 6B).

The H_2_O_2_ induced pH oscillations were unique from the glycolytic oscillations previously described. The period was much slower with a frequency peaked at 2.4 mHz (Fig. 6C), an order of magnitude slower than traditional glycolytic oscillations. In our experiments, we observed a maximum of 15% of the population exhibiting these slow oscillations, whereas ~90% could be induced into glycolytic oscillations. NADH oscillations could not be visualized during the slow pH oscillations despite the magnitude of pH oscillation was similar to the previously measured glycolytic oscillations. This data suggested these pH oscillations are a more general response to metabolic changes, and that the glycolytic pathway is not involved. Similar to the glycolytic oscillations, individual cells were unsynchronized across a population and displayed heterogeneity in oscillation initiation, termination, and durations. This data revealed a new class of oscillatory behaviors in *S. cerevisiae*, and suggested pH oscillations may contribute broadly to metabolic reprogramming.

## Discussion

NADH oscillations have been intensely studied at the population level for the past 60 years, resulting in a close match between experiments and theory. Our data are similar to previously described oscillations with the observations of (i) oscillating cytoplasmic NADH concentration, (ii) similar frequencies to cyanide induced oscillations, and (iii) increasing oscillation frequency in the presence of increasing concentrations of external glucose. Thus, we believe that the pH oscillations are occurring from changing flux through the glycolytic pathway. However, our data are also distinct from previous observations in that (i) sustained oscillations are induced without metabolic poisons, (ii) individual cells show heterogeneity and do not synchronize, (iii) oscillations can stop and restart in a single cell without addition of extra glucose, and (iv) immobilization under a hydrogel enhances the fraction of oscillating cells. Thus, our data add new insight to the mechanisms by which cells undergo glycolytic oscillations.

Using our sensitive absolute pH sensor, we were able to answer several longstanding questions about glycolytic oscillations. (1) Do single cells undergo sustained oscillations? Yes. Our data clearly show that single isolated cells can undergo oscillations, and that the dynamics in a population are highly heterogeneous. Additionally, single cells can stop and restart oscillations showing that oscillations can initiate even without changing external conditions. (2) Do oscillations arise under natural conditions? Yes. We imaged sustained oscillations upon glucose addition, without the need for cyanide. Yeast often experience changes in local nutrient concentration, and are also found immobilized in biotic substrates. Though we have not observed oscillations in the wild, our data show they could occur. (3) Do oscillations change cell phenotype? Yes. We show that oscillating cells have lower mitochondrial membrane potential and take longer to bud than genetically identical, non-oscillating cells in the same field of view. Though we did not establish a causal link between the glycolytic oscillations and the delayed bud time, we have shown that oscillating cells display slowed growth rates and are correlated with phenotypic heterogeneity in the population. In addition to addressing questions about glycolytic oscillations, the corresponding pH changes reveal new facets of oscillations that must be considered when considering metabolism.

To measure the pH oscillations, we modified the existing GFP based pH sensor, super ecliptic pHluorin. SEP is extremely sensitive, though it is unable to report absolute pH values due to variance in sensor expression. Genetically encoded ratiometric pH sensors can also report absolute pH, though they all require illumination with 405 nm light which induces substantial phototoxicity. Our new fusion sensor maintains the high sensitivity of SEP, but also enables ratiometric imaging to extract absolute pH values without the need for damaging UV light. Adjusting the fusion sensor composition could enhance FRET efficiency and increase the signal-to-noise ratio. Similar ratiometric sensors could be constructed with pHuji and GFP, though blue light photo-activation of pHuji^40^ could prove challenging.

The oscillations we observed reveal that cytoplasmic pH in *S. cerevisiae* is highly dynamic across several timescales, which has important implications in the context of glycolytic oscillations, but also for general mechanisms of cellular regulation. Several recent papers highlight the importance of cytoplasmic pH as a second messenger which can regulate transcription factor localization, GPCR activation, and PKA activity^8^. These pH oscillations, when viewed through the lens of dynamic calcium^41^ and kinase^42^ signaling in mammalian cells, could form the basis of frequency encoded signaling. This is further supported by the slow, H_2_O_2_ induced oscillations that were unaccompanied by changing NADH pools. On a more practical note, our data show that researchers studying any fluorescent biosensor in *S. cerevisiae* must explicitly account for pH, as every fluorophore is intrinsically pH sensitive. It remains a struggle to use fluorescent biosensors to measure additional cellular quantities due to the inherent pH dependence of fluorescent proteins.

Every enzyme has reaction kinetics tuned by the local pH, including a central regulator of glycolytic flux, phosphofructokinase (Pfk)^43^. Glycolytic oscillations are most often modeled as ATP/ADP allosteric regulation of Pfk, however, we show that pH may be able to tune the activity of Pfk, as well as every other enzyme in the cytoplasm with a pK_a_ in the range of 6.7–7.2. This is important in light of recent control theoretical analyses suggesting modulation of other enzymes, specifically pyruvate kinase, enhances robustness of glycolysis^13^. We propose that pH can act as a regulator of glycolytic flux and ATP consumption, through the activity of PMA. It is possible that the oscillations, can then act as a governor to slow the overall flux through glycolysis, and enable the cell to re-initiate glycolysis without being overwhelmed by ROS generated through respiration.

Our data show that pH oscillations arise during periods of metabolic transition in the population. Cells growing in log phase or in long term stationary phase did not oscillate upon glucose addition. When the population was undergoing diauxic shift, from glycolysis to respiration, glucose addition led to the initiation of pH oscillations. A similar switch that induces catabolism upon H_2_O_2_ challenge also induced pH oscillations with a much slower frequency. These data are reminiscent of oscillations in Akt activity in epithelial cells^42^ which modulate metabolic activity in higher eukaryotes. *S. cerevisiae* could be undergoing similar regulation exploiting cytoplasmic pH as the controlling mechanism. Overall, our data highlight the importance of single cell dynamic signaling, and open the door to deeper investigations into the role of pH dynamics in yeast.

## Materials and Methods

### Data availability

All raw and processed data will be made available to researchers upon request.

### Yeast strains and plasmids

Yeast Strain BY4741 was used for all wildtype experiments. The Vma2 knockout was obtained from the Euroscarf (MATa) haploid yeast knockout collection. Yeast strains containing plasmids expressing fluorescent sensors were created using a lithium acetate transformation. Yeast strains were grown in 2% glucose minimal media supplemented with –Leucine 100X amino acid dropout in a shaking 30 C incubator.

All plasmid cloning was performed in BW25113 *E. coli* before transformation into yeast. *E. coli* codon optimized SEP was PCR amplified for insertion into indicated plasmids. Ratiometric pHluorin, pHuji, and mRuby2 were constructed as a gBlock (IDT) using a *Saccharomyces cerevisiae* codon optimization. Mitochondrial matrix targeting was achieved by fusing the Su9 targeting sequence to the N-terminus of SEP-mRuby. Sensors were cloned into a 2-micron or centromeric plasmid (see Supplementary Table). All plasmids created for this publication will be available for distribution through Addgene.

### Preparing cells for imaging

Cells were typically immobilized under 1% agarose pads made with PBS (without calcium or magnesium), except for the long term budding experiments where pads were made with minimal medium without glucose. Cells were grown to an OD 600 of ~1.0, and from the 10 mL shaking culture, 100 μL of cell suspension was collected from and aliquoted into a 1.7 mL centrifuge. The cell suspension was left to settle or spun down at ~500 × g. 1–2 μL of the cell pellet was then placed on top of pad and allowed to settle onto the pad for at least 1 min. The pad with cells was then placed cell-side down into a glass bottom plate so that yeast were immobilized in a monolayer between the glass and agarose pad.

To image cells in a liquid droplet, cells grown to late-log and left in an Eppendorf tube for ~5 hours to induce oscillation. Cells were then washed in PBS and either placed onto an agarose pad or diluted. The diluted cells were placed onto a glass coverslip in a 150 μL droplet, and the cells sedimented on the coverslip after ~20 minutes. Glucose addition was achieved by gently placing a 1.5 μL drop of 10% glucose without inducing mixing. The presence of the glucose was seen by the increase in SEP fluorescence indicating an increase in cytoplasmic pH.

To add chemicals to the immobilized cells, a concentrated drop (~2.5 μL) was added to the top of the pad (~250 mg) and allowed to diffuse (Table 1). Glucose took ~5 minutes from to diffuse from the top to the cell layer. Unless otherwise noted, 3 uL of 10% Dextrose Minimal Media -Leu was added to initiate oscillations. Experiments evaluating effect of incubation inside of closed centrifuge tube were performed on five 100 ul aliquots that were removed from the shaking culture simultaneously. Cells were then drawn from each tube in 30 minute intervals to minimize accumulation disturbance within a single sample tube. Measurements of glucose in the supernatant were performed using a colorimetric glucose test strip (LW Scientific).

**Table 1:** List of chemical treatments on *S. cerevisiae*.

Chemical	Stock concentration	Diluted concentration	Supplier
Glucose	555 mM	5.5 mM	Sigma
Ebselen	10 mM	100 μM	Sigma
Sodium Orthovanadate	200 mM	2 mM	Sigma
H_2_O_2_	50 mM	500 μM	Sigma
Cyanide	500 mM	5 mM	Sigma

Each compound was added at 100× concentration on top of an agarose pad and allowed to diffuse down. The final, diluted concentration, is calculated by measuring the mass of the pad, and adding 1% of the 100× stock liquid on the top.

### Imaging conditions

Imaging took place on 3 different microscopes depending on the experiment.

For all experiments with SEP or SEP-mRuby (excepting the mitochondrial targeting) were taken on a Nikon NSTORM under high angle illumination. Lasers at 488 nm (SEP) and 561 nm (mRuby) were reflected off a quad-band dichroic mirror. A 60× water objective (NA 1.2) was imaged onto an EMCCD (Andor 897). Typical illumination intensities were 4025 mW/cm^2^ (488 nm) and 697 mW/cm^2^ (561 nm). Images were acquired sequentially by first illuminating with 488 nm alone and capturing, followed by 561 nm alone and capturing. Exposure was set to 100 ms for each color (200 ms total), and a new exposure was triggered every 2 seconds (200 ms illumination, 1.8 sec off). Imaging NADH used the same parameters, except a second dichroic was used (DAPI, 455/40 emission) with a 365 nm LED excitation from an Excelitas Xcite at 6250 mW/cm^2^. The software was configured to acquire the NADH images at 2 × 2 binning, while keeping the SEP and mRuby images at 1 × 1 binning.

The long term budding experiments and the time in tube experiments were conducted on a Nikon TiE with environmental controls (OkoLabs) set to 30 °C. A 40× air objective (NA 0.95) was imaged onto a Hamamatsu Flash 4 sCMOS camera. Light from a SpectraX LED source was reflected from a quadband dichroic and imaged through a 568 LP emission filter. Illumination was achieved with a 565/20 excitation filter at an intensity of 2178 mW/cm^2^. Images were acquired with 100 ms of exposure every 2 seconds (100 ms on, 1.9 seconds off) to image oscillations. To watch long term growth, images were acquired every 15 seconds (100 ms on, 14.9 seconds off).

Movie imaging mitochondrial targeting (TMRM or SEP), SNARF-5F-AM, and ratiometric pHluorin were conducted on a Nikon spinning disk confocal microscope. A 100× oil objective (NA 1.45) was illuminated with lasers through the Nipkow disk and imaged onto an EMCCD (Andor iXon Ultra 888). Excitation of SEP used a 488 nm laser at 1090 mW/cm^2^ and TMRM was illuminated with a 561 nm laser at 692 mW/cm^2^. Exposures were 100 ms at each color, followed by 1.8 seconds of rest for a measurement frequency of 0.5 Hz. Excitation of SNARF-5f-AM was accomplished using a 488 nm laser, an exposure time of 300 ms, and a 620/60 nm emission filter. Excitation of ratiometric pHluorin was accomplished using 488 nm laser, 100 ms exposures, and a 520/50 nm emission filter.

### Absolute pH sensor and quantification

To calibrate the SEP-mRuby absolute pH scale, cells were immobilized in a flow cell using concanavilin (Sigma). Cells were permeabilized with 0.1% w/v digitionin PBS solution for 5 minutes. McIlvaine’s buffers were used to create external pHs from 5.5 to 8.5. For every pH, the buffer was flowed through the flow cell for 60 seconds followed by acquisition of 5 images with both 488 nm and 561 nm illumination. These movies were then averaged to acquire a SEP to mRuby ratio at each external pH.

To test for potential FRET between SEP and mRuby in the fusion sensor, we measured the amplitude of oscillations using 488 nm excitation using both a GFP emission filter (515/30) and a TRITC emission filter (620/60) for both SEP alone and the SEP-mRuby fusion. Measuring the bleed-through amplitude of SEP directly into the TRITC channel, it was calculated that at the highest pH, ~8% of the SEP signal was lost due to FRET. This loss does not affect the calibration curve generated above as the ratio was taken using 561 nm excitation of mRuby which is independent of FRET. It does, however, reduce the dynamic range of the sensor.

To extract the best fit parameters to a hill curve, each cell in the field of view was segmented, and the ratio of SEP to mRuby was extracted at each external pH. The response of each individual cell was then fit to a hill curve. All the fit pKa were then plotted on to a histogram, which was then fit to a Gaussian, which yielded a best population fit with a pKa of 7.4.

### TMRM staining

TMRM (ThermoFisher) was added to cells as per the manufacturer’s directions. Stock solutions were made at a concentration of 1 mM in DMSO. To load cells, the stock solution was diluted 1:200 in a suspension of cells to a final concentration of 5 μM. Cells were incubated with TMRM for 105 minutes at room temperature without shaking before placement on an agarose pad. Mitochondrial staining was imaged on a spinning disk confocal microscope (see above).

### SNARF-5F-AM staining

A 1 mM solution of SNARF-5F 5-(and 6)-Carboxylic Acid, Acetoxymethyl Ester, Acetate (ThermoFisher #S23923) by resuspending in DMSO. A 100 μl aliquot of cell suspension was collected, washed with PBS, and resuspended in 100 μl PBS. SNARF5F solution was add to 100 μl of cell suspension at a final concentration of 10 μM and incubated at room temperature for >30 minutes. Subsequent preparation for imaging cells on an agarose pad followed procedures describe above. Image acquisition conditions are described above.

### Flow cell

Flow cells were constructed from 2 mm thick silicone, cut to form a channel. The silicone channel was pressed between a coverslip which was used to adhere yeast via concanavalin, and a glass slide with two holes drilled 15 mm apart and 18 gauge needles adhered. Glucose containing buffers were flowed through this chamber at a rate of 100 µl/s. To control the oscillating buffer conditions, an Arduino (Arduino One) controlling two solenoid pinch valves alternated between 10 s closed, 10 s open state in order to flow media through 1/32” ID Tygon Tubing from the reservoir into the flow cell chamber. This setup resulted in an experiment where every 20 seconds the medium pH was switched between 5.2 and 7.6.

### Image processing and data analysis

All data analysis was performed with custom scripts in Matlab (Mathworks, R2016a). All scripts will be distributed to researchers upon request.

#### Segmentation

Movies were first aligned to compensate for any drift using the *imregister* command in Matlab. Cells were segmented by taking the average image in time, subtracting a dilated image to create rings marking each cell and finally identifying circles using *imfindcircles* with inputs for the magnification that qualitatively fit the size distribution of cells in a movie.

Mitochondria were segmented by first segmenting the cells as above, and then identifying bright regions in the bounding box for each identified cell. Mitochondria were labeled as having an intensity above a user set threshold that qualitatively matched the movies. For each cell, a movie was generated showing the raw fluorescence and the mask of intense pixels. The mitochondrial trace was then calculated as the mean of all pixels within this mask. If there were no pixels above the threshold, the program returned an empty value.

#### Identifying oscillating cells

Oscillating cells were identified in time by using the *spectrogram* command in Matlab. Points in time that were flagged as oscillating had to meet the criteria of rising above 3.5× the standard deviation of low points in the power spectrum. Any peak in the power spectrum that met that criteria was then marked as an oscillating point in time, and the frequency was calculated by finding the frequency of the peak maximum. At every time point in the spectrogram the presence of oscillations and the frequency were calculated for each cell. The frequency for each cell plotted in histograms is then the mean of all the peak frequencies that were identified for each trace.

#### Calculating phase difference between two signals

The relative phase between two oscillating signals was calculated by first truncating oscillating intensities to the timepoints marked as oscillating by the spectrogram (see above). For each trace (eg. pH and NADH), the Hilbert transform was calculated using the *hilbert.m* command. The relative phase difference was then calculated using the *angle.m* command of H_1_(t)/H_2_(t).

## Electronic supplementary material

Supplementary Information

Supplementary Movie 1
