# Supplementary material for: Live Cell Imaging Reveals pH Oscillations in Saccharomyces cerevisiae During Metabolic Transitions
Source: Sci Rep. 2017 Oct 24;7:13922. doi: 10.1038/s41598-017-14382-0 (PMC5654966; doi:10.1038/s41598-017-14382-0)
Supplement: Supplementary file 1 — Supplementary Information [file 41598_2017_14382_MOESM1_ESM.pdf]

Benjamin J. T. Dodd[1] and Joel M. Kralj[1,2*]
[1]BioFrontiers Institute, and
[2]Molecular Cellular and Developmental Biology Department
University of Colorado – Boulder, 80303

[*]Corresponding author (joel.kralj@colorado.edu)

Live Cell Imaging Reveals pH Oscillations in *Saccharomyces cerevisiae* During Metabolic Transitions

Supplementary Information

SUPPLEMENTARY FIGURES

Figure S1 –

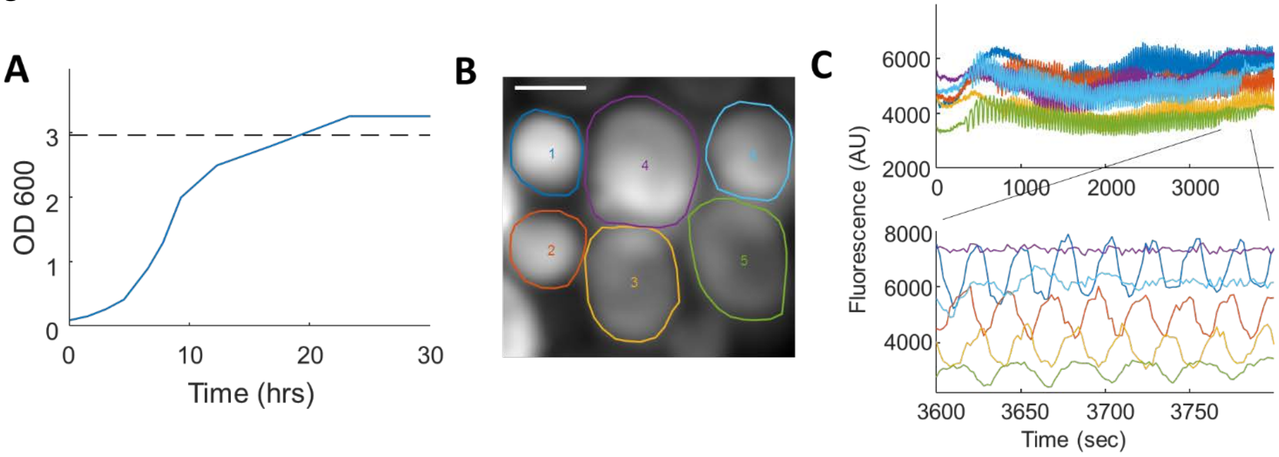

Figure S1 - (A) Growth curve for yeast cells grown in leucine drop-out medium. Oscillating cells were typically seen at an OD of ~2-3. (B) Individual cells expressing SEP with manually selected ROIs. Scale bar is 3 μm. (C) Time traces of cells from the same ROI of (B). Each cell oscillates with varying frequency and phase. The phase of each oscillator does not synchronize within 1 hour of imaging (zoomed region).

Figure S2 –

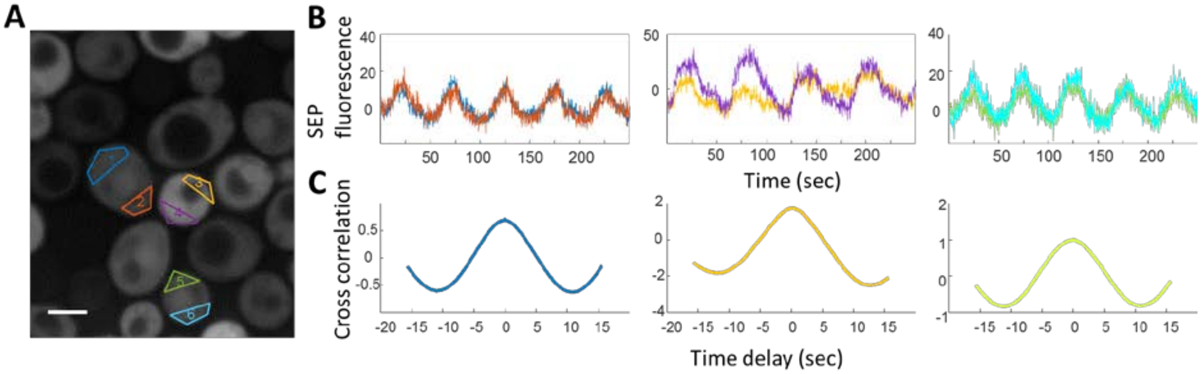

Figure S2 – (A) Average image of SEP expressing cells taken on a spinning disk microscope with 100x objective. Manually selected ROIs on opposite halves of the same cell are represented with different color lines. Scale bar is 2 μm. (B) Time traces taken at 10 Hz from the corresponding ROI in (A). (C) Cross correlation of each trace taken in B. The time lag between traces for each cell was at 0 seconds.

Figure S3 -

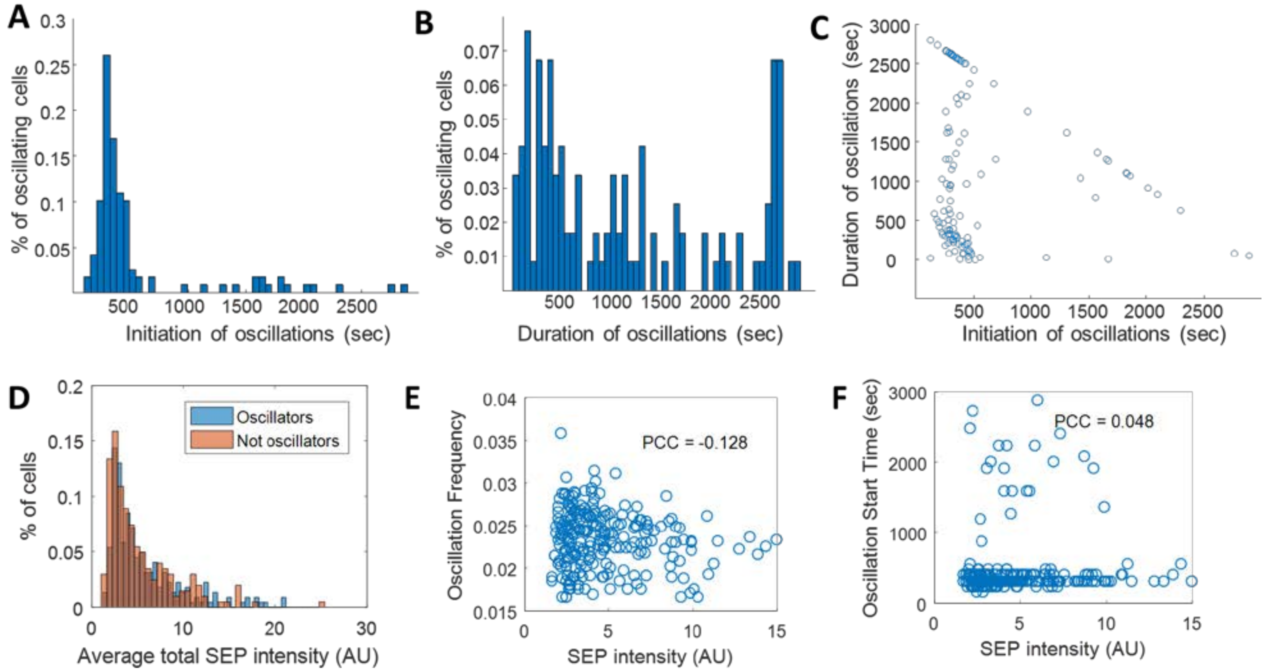

Figure S3 - (A) Distribution of the initiation of oscillations after the addition of glucose. (B) Distribution of the duration of oscillations after the addition of glucose. (C) Scatter plot showing the initiation of oscillation as compared to the duration. All cells along the diagonal were oscillating until the end of the recording. (D) Histograms of initial SEP intensity for both oscillators (blue bars) and non-oscillators (red bars). (E) Scatter plot showing low correlation between the mean SEP intensity and the oscillation frequency. (F) Scatter plot showing low correlation between the mean SEP intensity and the oscillation start time.

Figure S4 –

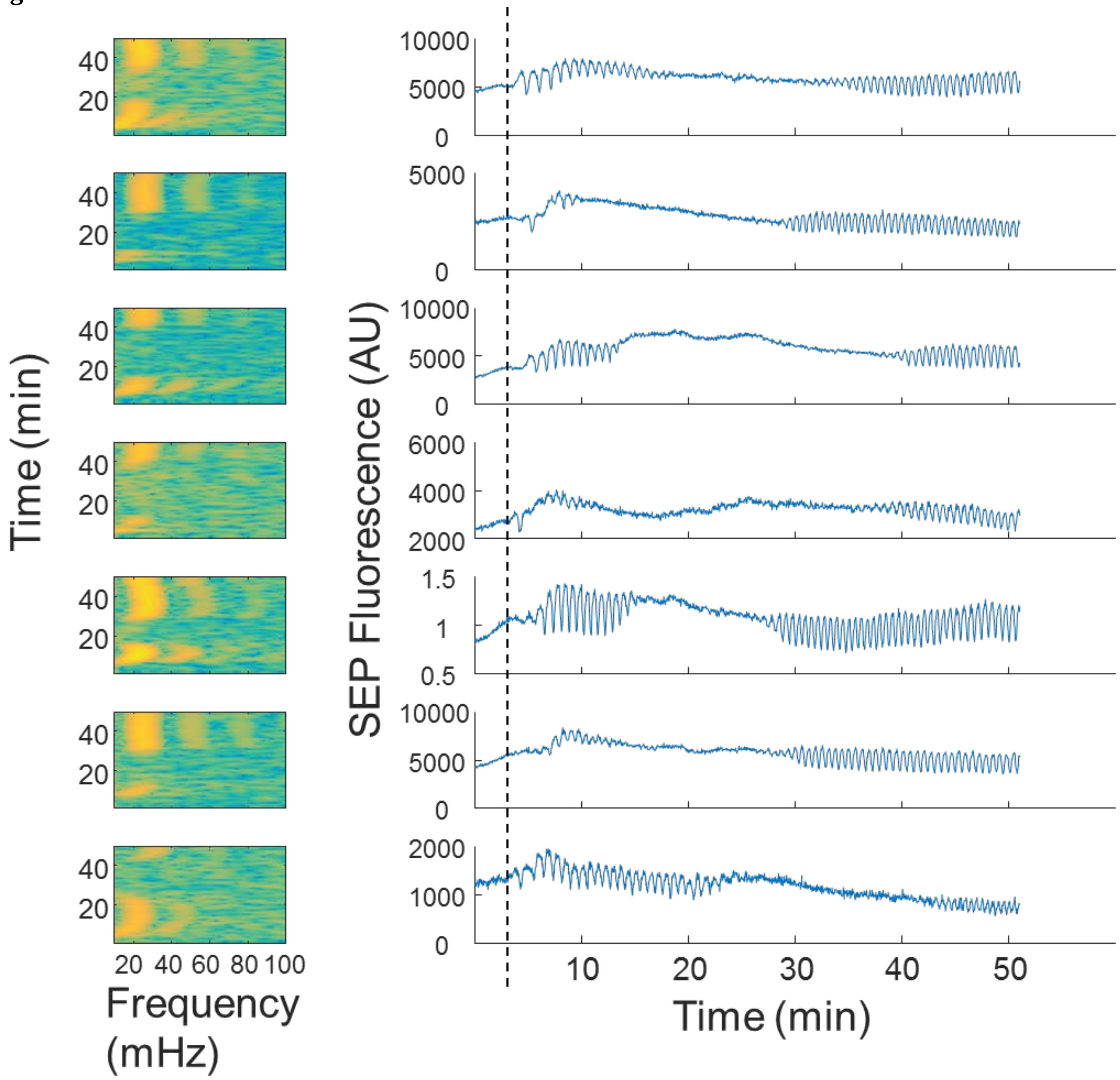

Figure S4 - Examples of single cells starting oscillations after glucose addition, stopping, and then restarting. (Left) Spectrograms of oscillators and (right) the corresponding time trace. Cells were imaged expressing cytoplasmic SEP. The dashed black line indicates the addition of glucose.

Figure S5 –

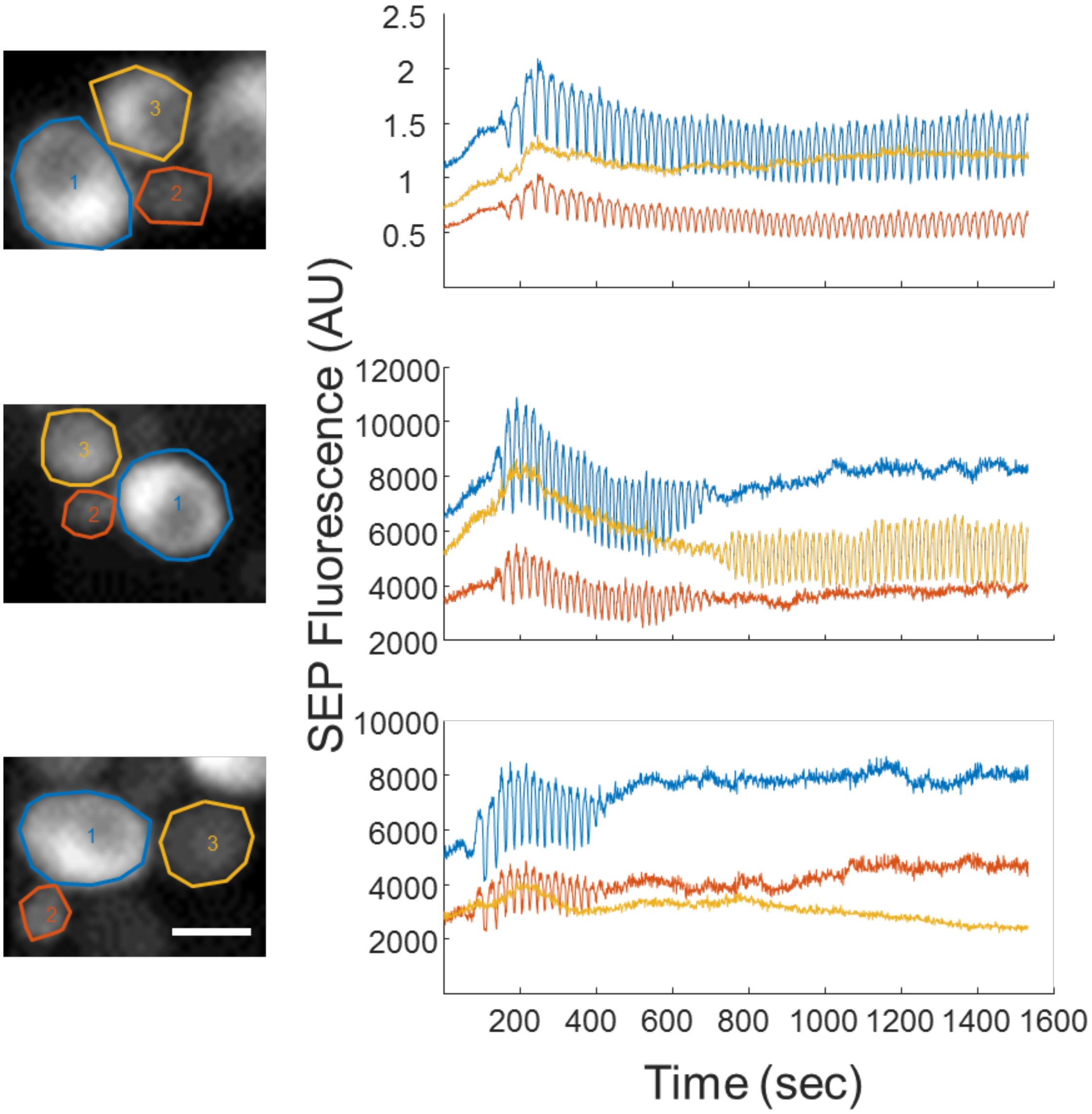

Figure S5 - Examples of budded yeast cells with oscillating pH. (Left) Image of cells expressing SEP with manually selected ROIs. Scale bar is 3 μm. Blue – mother cell, red – daughter bud, yellow – neighboring cell. (Right) Time traces associated with each ROI in the image. The blue and red traces are in phase, and stop and start at the same time, and are uncorrelated with the activity of the neighboring cell (yellow trace).

Figure S6 –

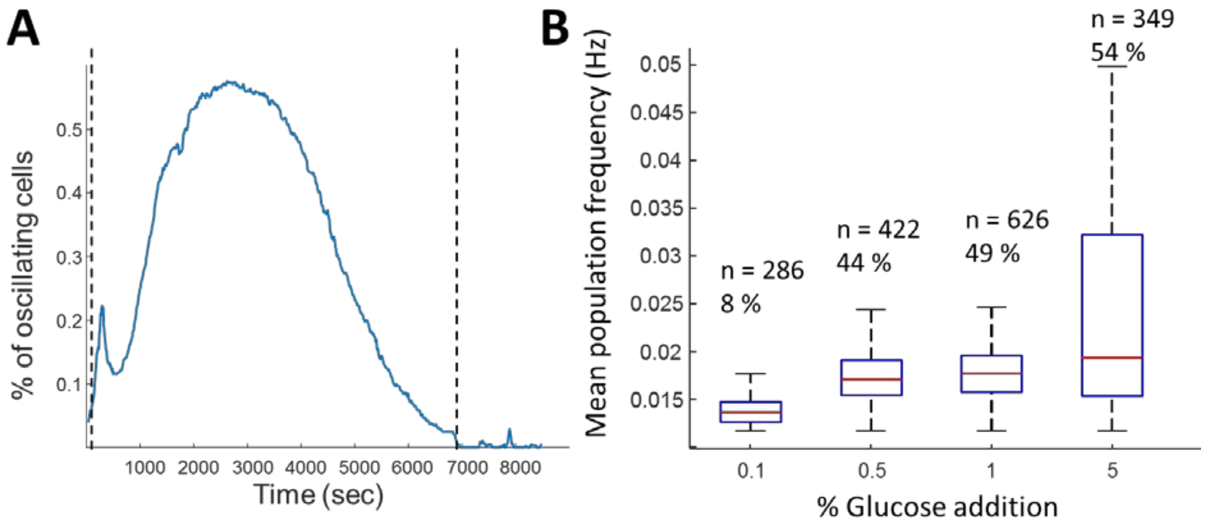

Figure S6 – (A) Oscillations only begin during transition from starvation to glucose. Figure shows the percentage of oscillating cells as a function of time after glucose addition (dashed black line). Upon re-addition of glucose after 2 hours, no cells started oscillating indicating these oscillations only occur with specific pre-treatment of cells. (B) Box plots showing a change in population median frequency with increasing external glucose concentration. The median concentration increases 70% from .0139 to .0197 Hz. Red line indicates median, box indicates 75/25%, and black dashed lines indicate 90/10% of the maxima. Outliers were analyzed but not shown. The number of cells for each box and the fraction of oscillating cells is shown above.

Figure S7 –

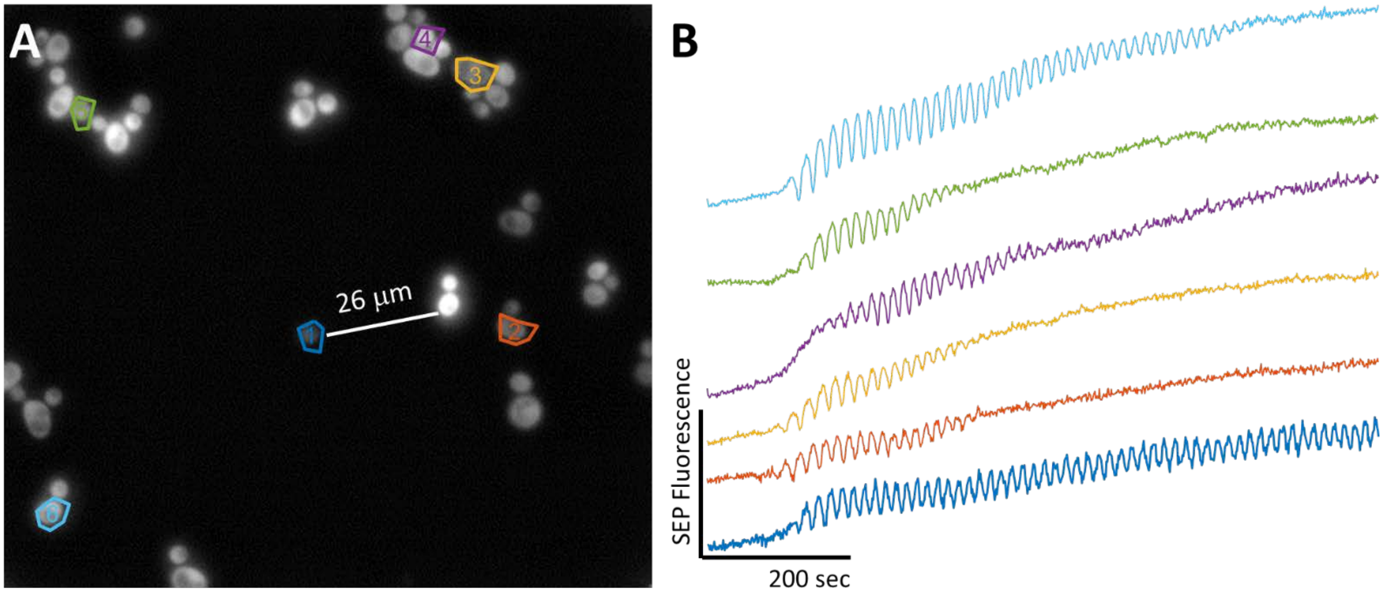

Figure S7 - (A) Image of sparsely plated cells expressing cytoplasmic SEP. Each color represents a user selected ROI. Cell marked with a dark blue line (1) is 26 μm from the closest neighboring cell. (B) Time traces from each of the corresponding ROIs in (A). The cell marked with the dark blue line shows pH oscillations, as do many of the distant neighbors.

Figure S8 –

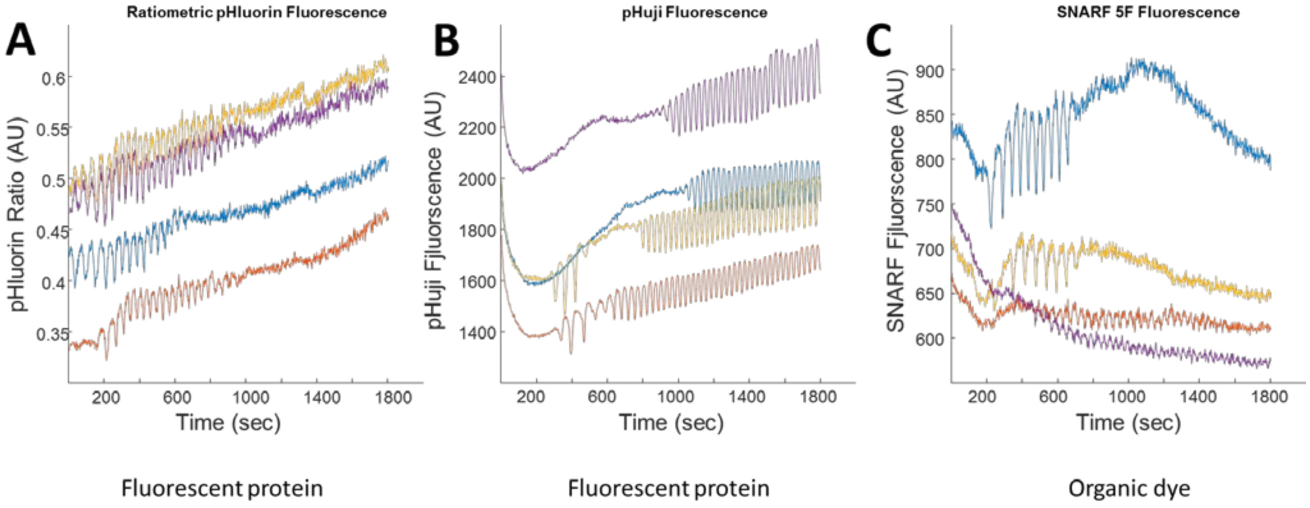

Figure S8 - Examples of cells showing pH oscillations with different pH indicators (A) ratiometric pHluorin (genetically encoded), (B) pHuji (genetically encoded), and (C) SNARF-5F-AM (organic dye).

Figure S9 –

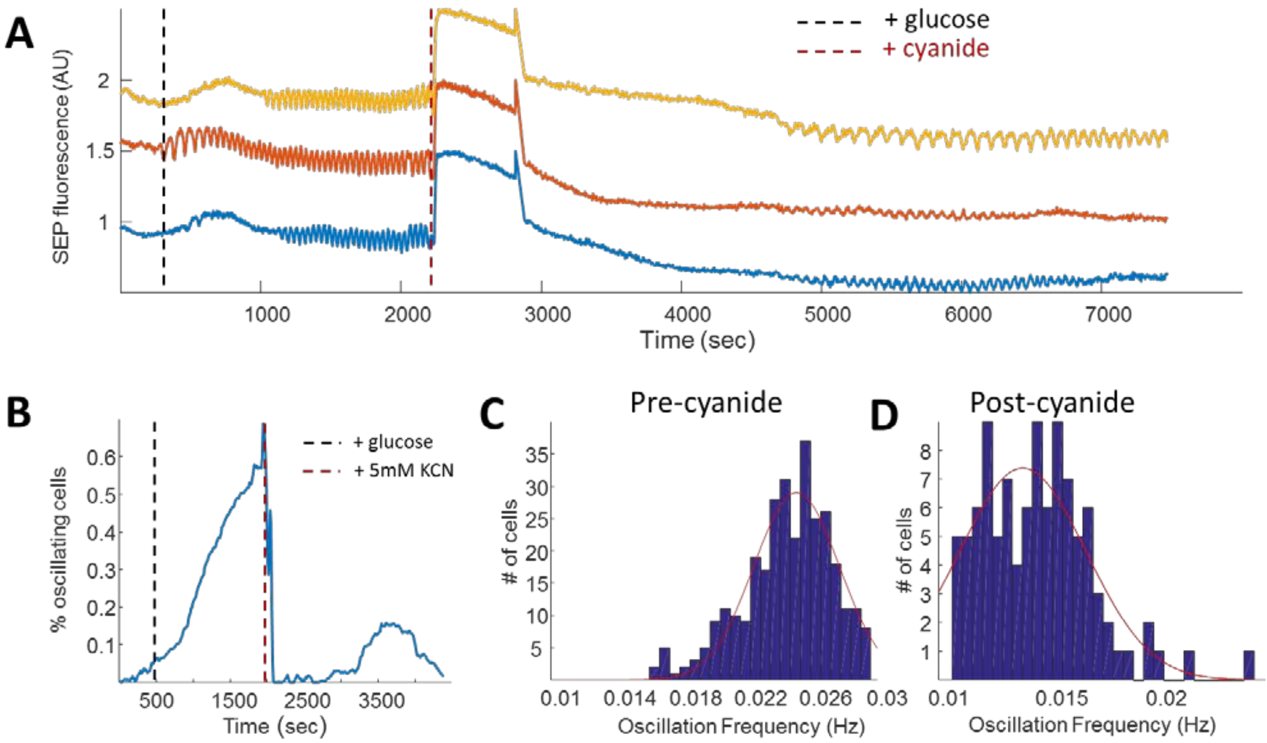

Figure S9 - Addition of cyanide to oscillating cells. (A) Example traces of cells that showed oscillations before and after addition of cyanide. Glucose addition (black dashed line) caused cells to start oscillating. Cyanide addition (red dashed line) induced an increase in cytoplasmic pH. Upon recovery, some cells re-started oscillation, though the amplitude was smaller and they were not synchronized. (B) Percentage of oscillating cells measured as a function of time from the same experiment as (A). (C & D) Distribution of oscillation frequencies before and after cyanide addition.

Figure S10 –

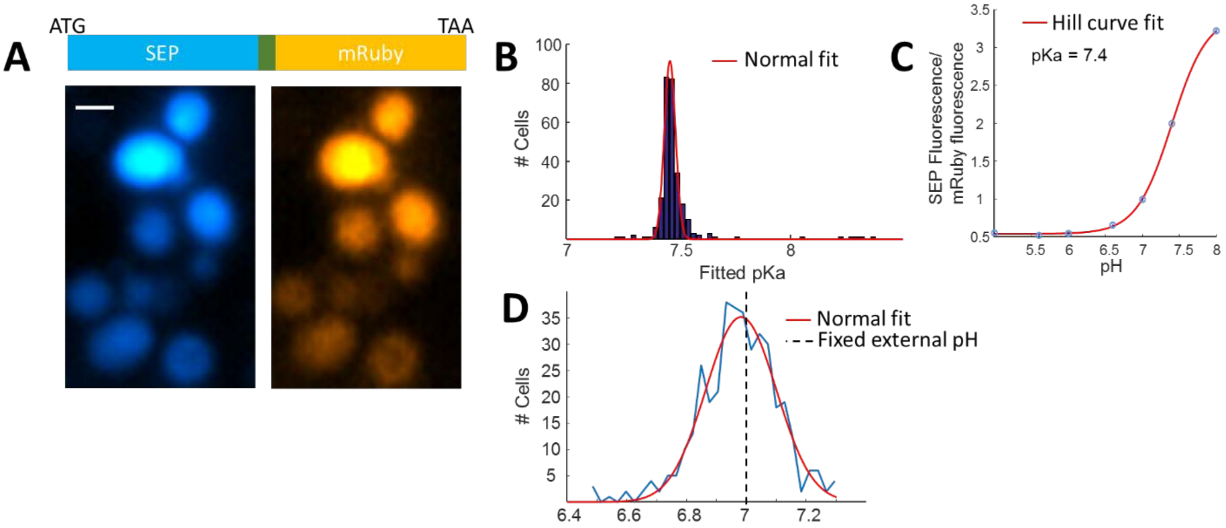

Figure S10 - (A) Construction of a ratiometric pH sensor using a fusion of SEP and mRuby3. Images showing illumination with 488 nm (SEP) and 561 nm (mRuby). (B) To calibrate the sensor, each cell had the SEP to mRuby ratio calculated across a range of pHs while cells were permeabilized with digitonin. Each cell was fit to a hill curve which yielded a unique pKa. Each pKa was plotted in a histogram which was fit to normal distribution with a population pKa of 7.4. (C) Data points for the population (blue dots) and the hill curve fit (red line) to a pKa of 7.4. (D) A new set of cells was permeabalized and set to pH 7.0. Each cell was then fit using the parameters generated in B and the apparent pH was plotted as a histogram. The histogram was fit to a normal distribution with a pH of 6.98 with a half width half maximum of 0.18 pH units.

Figure S11 –

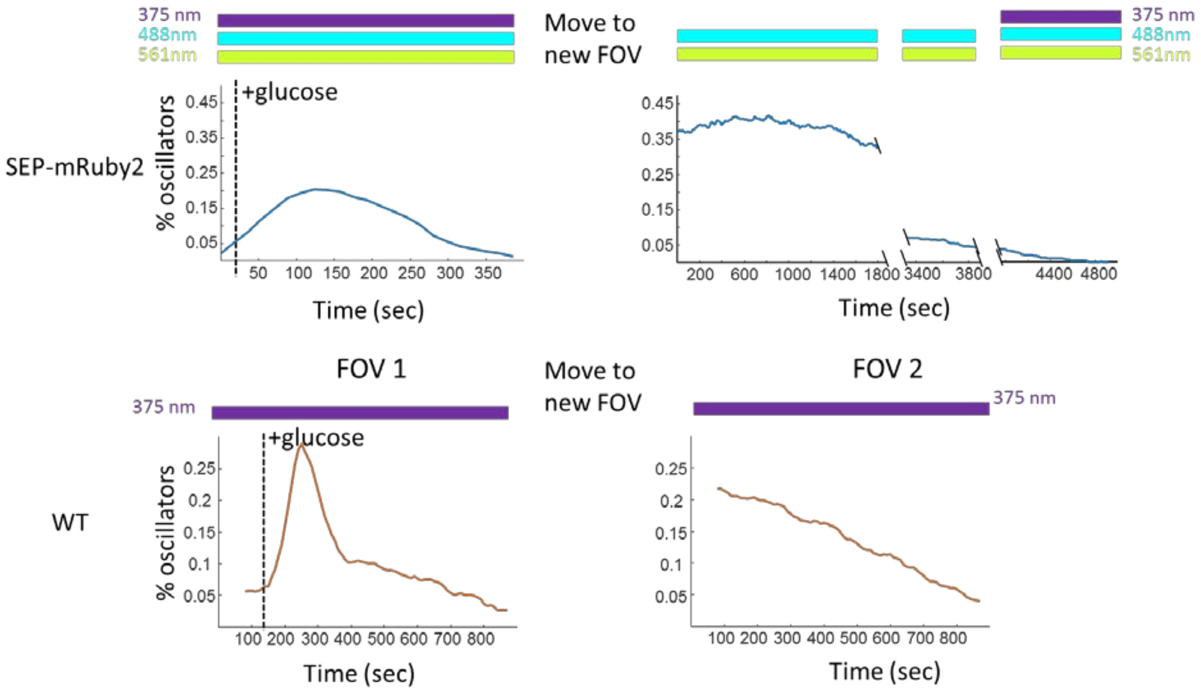

Figure S11 - UV light suppresses pH oscillations. (Top) The fraction of oscillating cells was calculated for a population while simultaneously imaging SEP-mRuby and NADH autofluorescence. The fraction had been reduced to < 5% within 5 minutes after glucose addition. Illumination of a new field of view of the same pad showed that 40% of the cells were still oscillating when imaging with 488 nm and 561 nm light. Cells continued to oscillate in the new field of view, reducing to 7% after 1 hour. However, the same field of view exposed to UV eliminated all oscillators within 5 minutes. (Bottom) Non-expressing cells showed oscillations that were also suppressed by UV light. Cells imaged with NADH autofluorescence initially showed a high fraction of oscillating cells that quickly died down. A new field of view had high numbers of oscillators that also decreased with time.

Figure S12 –

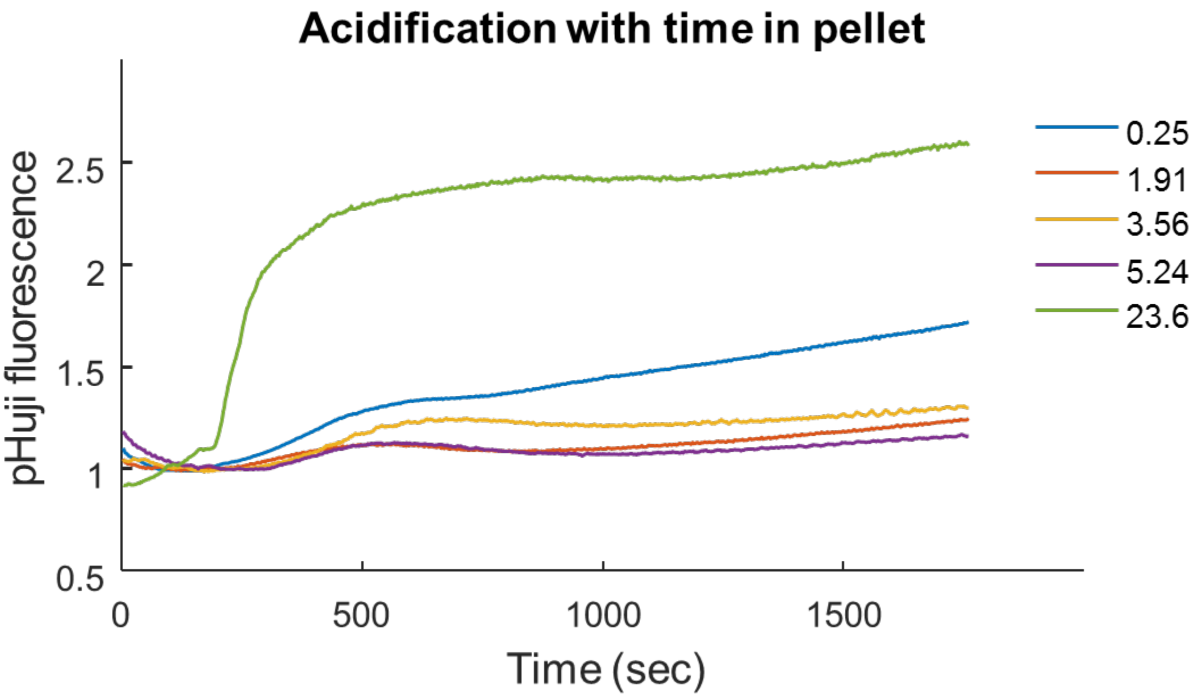

Figure S12 – Increase in baseline fluorescence change upon glucose addition with increased time in the Eppendorf tube. The longer the cells remained sedimented in the pellet, the larger the increase in baseline fluorescence indicating an increased acidification of the cytoplasm. Data shown is the mean time trace of all cells in a field of view (> 300 for each condition).

Figure S13 -

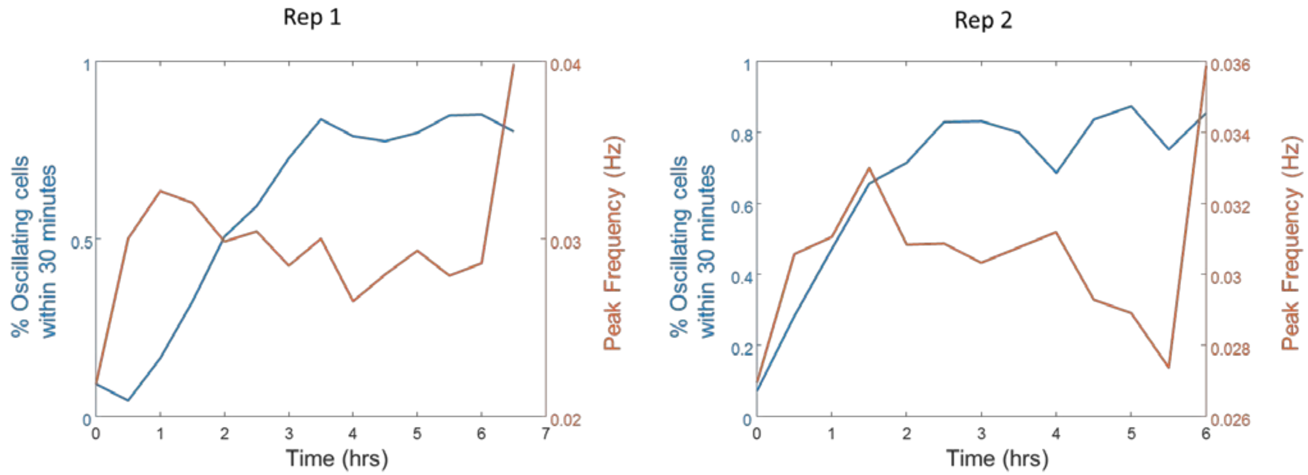

Figure S13 - Cells that are spun down in an Eppendorf tube show similar oscillation behaviors to cells left to settle without spinning. Two replicates of the time in tube experiment showing an increase in the fraction of oscillating cells with increased time incubating in the tube.

Figure S14 –

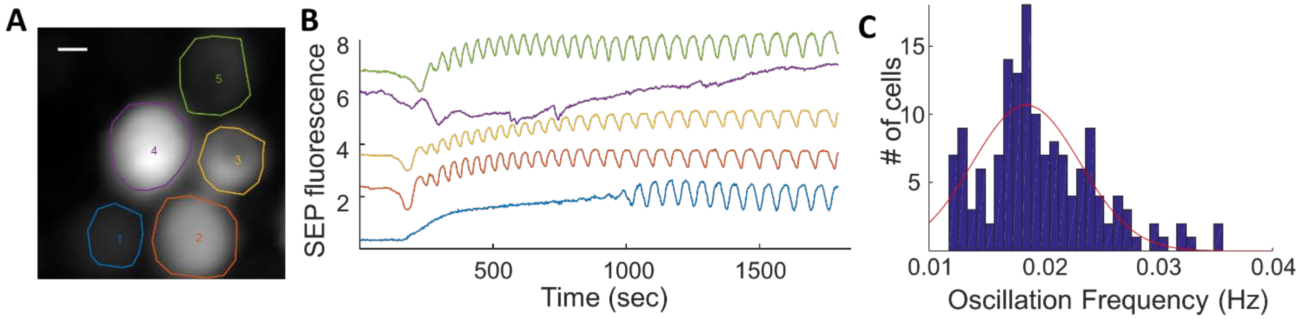

Figure S14 - Yeast cells taken directly from a colony grown on agarose show oscillations. (A) Image of cells expressing SEP taken directly from a colony and placed on an agarose pad. Each color represents a manually selected ROI. Scale bar is 1 μm. (B) Time trace of cells from (A) showing pH oscillations from cells taken directly from a colony and fed glucose. (C) Distribution of oscillation frequencies of cells taken directly from an agar plate.

Figure S15 –

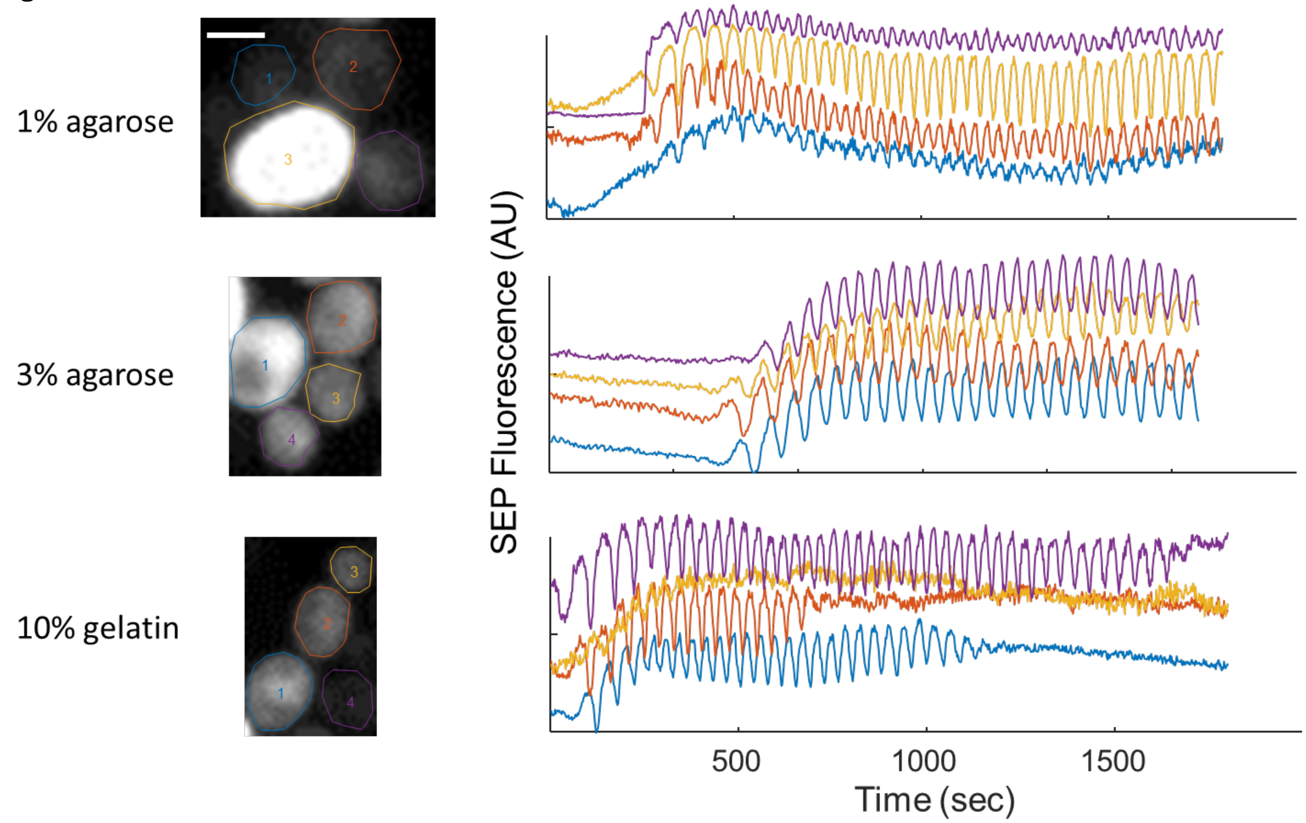

Figure S15 - The hydrogel substrate does not dictate the presence of oscillations. (Left) Images and manually selected ROIs for cells immobilized under a 1% agarose pad (typical), 3% agarose pad, and 10% gelatin pad. Each pad was made with PBS at the noted concentration. (Right) Time traces from each ROI showing yeast cells undergo pH oscillations under each hydrogel. Scale bar is 2 μm.

Figure S16 –

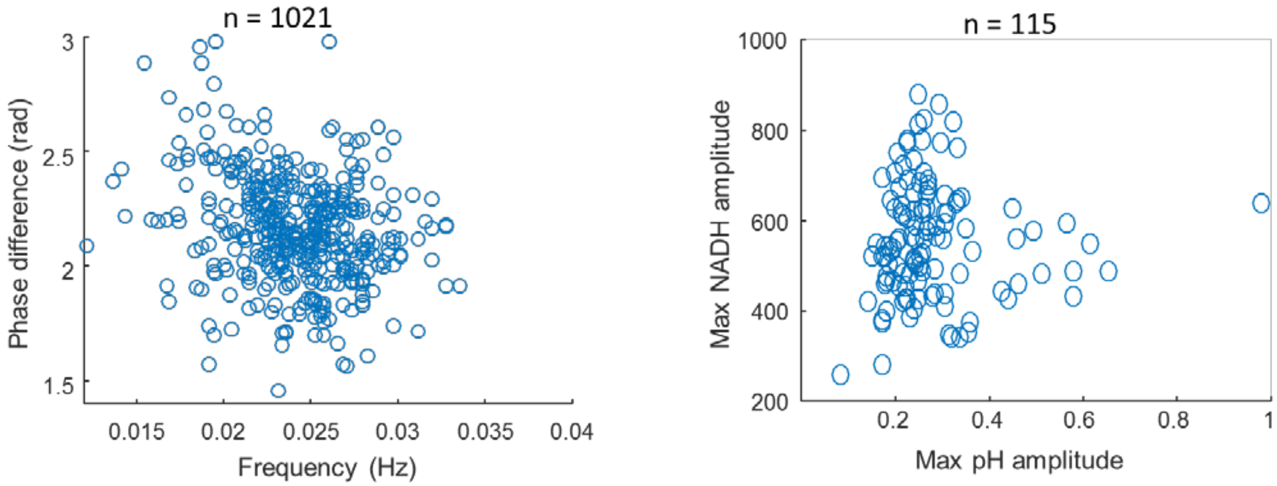

Figure S16 - (A) Scatter plot of individual cells imaged with SEP-mRuby and NADH autofluorescence simultaneously. The frequency of each cell is plotted on the x-axis and the phase difference between the pH and NADH oscillations on the y-axis. (B) Under the same imaging conditions as (A), a scatter plot showing the maximum pH amplitude on the x-axis and the maximum NADH amplitude on the y-axis.

Figure S17 –

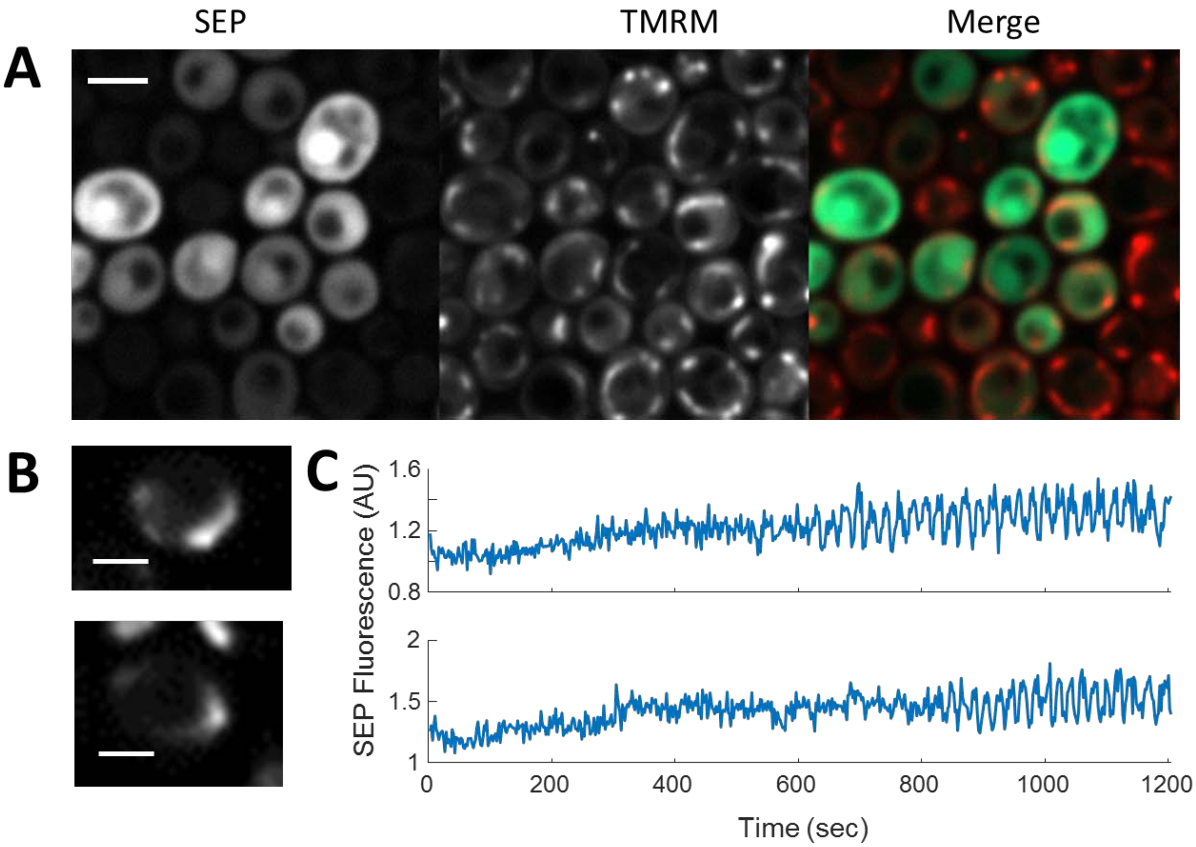

Figure S17 – Mitochondrial matrix pH oscillates in cells undergoing cytoplasmic pH oscillations. (A) Images of yeast cells expressing cytoplasmic SEP and incubated with the membrane permeable dye TMRM. SEP (imaged at 488 nm) and TMRM (imaged at 561 nm) do not show any spectral cross talk, and the TMRM clearly stains mitochondria. Scale bar is 3 µm. (B) Images of yeast cells expressing a mitochondrially targeted SEP. Scale bar is 2 µm. (C) Time traces of each cell from (B) showing mitochondrial pH oscillates after the addition of glucose, similar to the cytoplasmic pH.

Figure S18 –

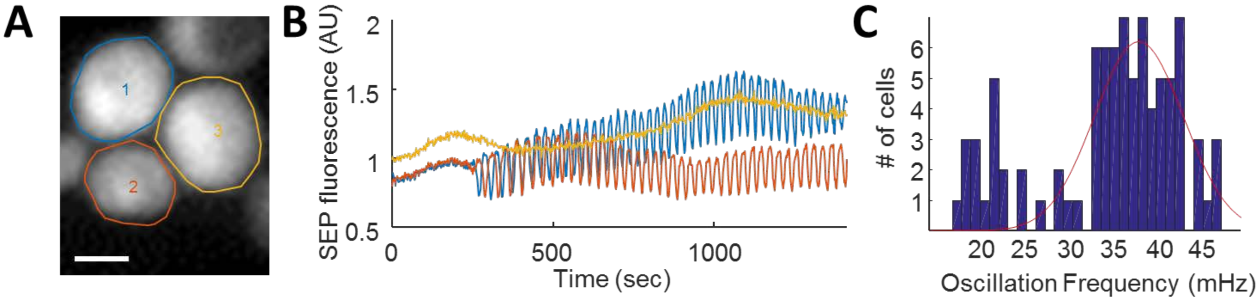

Figure S18 - (A) Image of Vma2KO cells expressing SEP with manually selected ROIs. Scale bar is 3 μm. (B) Time traces of ROIs from the selected regions in (A). (C) Frequency distribution of oscillating cells containing a Vma2 KO.

Figure S19 –

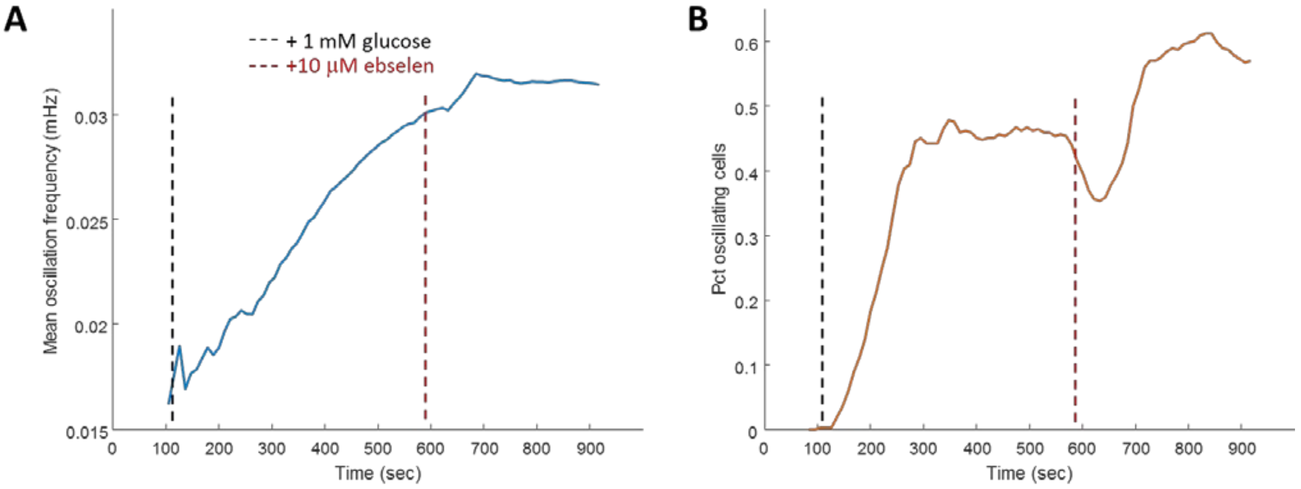

Figure S19 – (A) Addition of 10 µm ebselen caused an increase in the oscillation frequency. Data shown is the mean frequency of all oscillating cells in a FOV. (B) The percentage of oscillating cells increased upon addition of 10 µm ebselen.

Figure S20 –

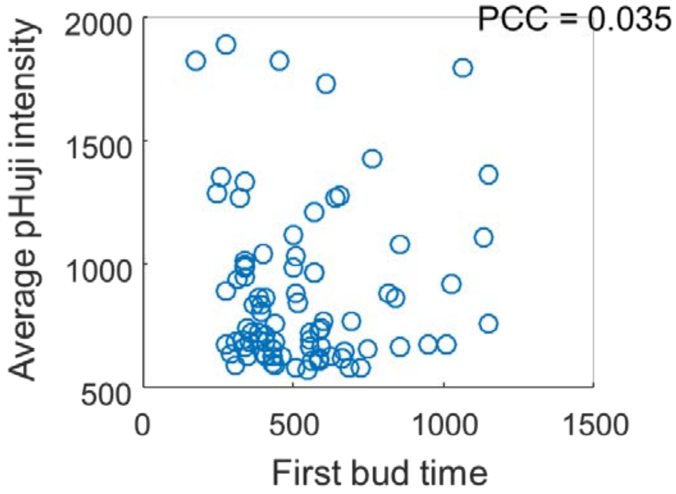

Figure S20 – Scatter plot comparing the mean pHuji intensity to the first appearance of a bud for individual cells. There is low correlation between the mean pHuji intensity and the time to bud.
